# Performance of three delignifying pretreatments on hardwoods: hydrolysis yields, comprehensive mass balances, and lignin properties

**DOI:** 10.1186/s13068-019-1546-0

**Published:** 2019-09-09

**Authors:** Aditya Bhalla, Charles M. Cai, Feng Xu, Sandip K. Singh, Namita Bansal, Thanaphong Phongpreecha, Tanmoy Dutta, Cliff E. Foster, Rajeev Kumar, Blake A. Simmons, Seema Singh, Charles E. Wyman, Eric L. Hegg, David B. Hodge

**Affiliations:** 1 Department of Biochemistry & Molecular Biology, Michigan State University, East Lansing, MI 48824 USA; 2 DOE Great Lakes Bioenergy Research Center (GLBRC), Michigan State University, East Lansing, MI 48824 USA; 3 Department of Chemical and Environmental Engineering, University of California, Riverside, CA USA; 4 BioEnergy Science Center (BESC) and Center for Bioenergy Innovation (CBI), Oak Ridge National Laboratory, Oak Ridge, TN 37831 USA; 5 Joint BioEnergy Institute (JBEI), Lawrence Berkeley National Laboratory, Berkeley, CA 94720 USA; 6 Chemical & Biological Engineering Department, Montana State University, Bozeman, MT 59715 USA; 7 Department of Chemical Engineering and Materials Science, Michigan State University, East Lansing, MI 48824 USA; 8 Division of Sustainable Process Engineering, Luleå University of Technology, Luleå, Sweden

**Keywords:** Pretreatment, Cellulosic biofuels, Lignin, Aromatic monomers

## Abstract

**Background:**

In this work, three pretreatments under investigation at the DOE Bioenergy Research Centers (BRCs) were subjected to a side-by-side comparison to assess their performance on model bioenergy hardwoods (a eucalyptus and a hybrid poplar). These include co-solvent-enhanced lignocellulosic fractionation (CELF), pretreatment with an ionic liquid using potentially biomass-derived components (cholinium lysinate or [Ch][Lys]), and two-stage Cu-catalyzed alkaline hydrogen peroxide pretreatment (Cu-AHP). For each of the feedstocks, the pretreatments were assessed for their impact on lignin and xylan solubilization and enzymatic hydrolysis yields as a function of enzyme loading. Lignins recovered from the pretreatments were characterized for polysaccharide content, molar mass distributions, β-aryl ether content, and response to depolymerization by thioacidolysis.

**Results:**

All three pretreatments resulted in significant solubilization of lignin and xylan, with the CELF pretreatment solubilizing the majority of both biopolymer categories. Enzymatic hydrolysis yields were shown to exhibit a strong, positive correlation with the lignin solubilized for the low enzyme loadings. The pretreatment-derived solubles in the [Ch][Lys]-pretreated biomass were presumed to contribute to inhibition of enzymatic hydrolysis in the eucalyptus as a substantial fraction of the pretreatment liquor was carried forward into hydrolysis for this pretreatment. The pretreatment-solubilized lignins exhibited significant differences in polysaccharide content, molar mass distributions, aromatic monomer yield by thioacidolysis, and β-aryl ether content. Key trends include a substantially higher polysaccharide content in the lignins recovered from the [Ch][Lys] pretreatment and high β-aryl ether contents and aromatic monomer yields from the Cu-AHP pretreatment. For all lignins, the ^13^C NMR-determined β-aryl ether content was shown to be correlated with the monomer yield with a second-order functionality.

**Conclusions:**

Overall, it was demonstrated that the three pretreatments highlighted in this study demonstrated uniquely different functionalities in reducing biomass recalcitrance and achieving higher enzymatic hydrolysis yields for the hybrid poplar while yielding a lignin-rich stream that may be suitable for valorization. Furthermore, modification of lignin during pretreatment, particularly cleavage of β-aryl ether bonds, is shown to be detrimental to subsequent depolymerization.

## Introduction

Lignocellulosic biomass represents an enormous reservoir of reduced carbon that offers the potential to serve as a feedstock for the production of renewable fuels, chemicals, and polymeric materials [1]. Furthermore, the adoption of these biomass-derived products can support outcomes that include increasing domestic energy security, reducing greenhouse gas emissions, and supporting domestic rural economies [2]. A diverse range of biomass-to-biofuels technologies is available, and in recent years commercial-, demonstration-, and pilot-scale processes for the deconstruction and conversion of the structural polysaccharides within lignocellulosic biomass to ethanol have been built [3]. These processes are based on herbaceous feedstocks (e.g., corn stover/fiber, sugarcane bagasse, wheat straw) and an acidic or mild alkaline hydrothermal pretreatment followed by enzymatic hydrolysis and fermentation of the hydrolysates to ethanol. The process-derived lignins are burned in a boiler to generate steam and electricity. Established technical and economic challenges to the widespread commercial deployment of these cellulosic biofuels processes include (1) substantially higher capital and operating costs relative to the starch- or sucrose-derived ethanol [4], (2) the supply chain challenges associated with the low bulk density, storage, and year-round availability of herbaceous feedstocks [5], (3) challenges with process integration (e.g., feedstock handling, fermentation inhibitors, etc.), and (4) the low carbon mass efficiencies of these processes, whereby 100 kg of dry biomass may yield up to 30-kg ethanol biofuel as the theoretical upper limit set by composition, hydrolysis yields, and fermentation yields.

While current processes utilizing pretreatment and enzymatic hydrolysis for the generation of cellulosic sugars employ herbaceous feedstocks, woody biomass offers several potential benefits as a bioenergy feedstock. Although exhibiting higher recalcitrance than herbaceous feedstocks, woody biomass has benefits that include high biomass productivities, high bulk densities relative to herbaceous feedstocks facilitating transportation and storage, year-round availability, and suitability for widespread cultivation on land that may be economically marginal for other agriculture uses [6]. Plantation-growth hybrid poplar and *Eucalyptus* ssp. are promising feedstocks for fiber and fuels [6–8] and have been proposed to be grown on production cycles ranging from 5 to 20 years [9]. Substantial research has been devoted to short-rotation woody crops such as hybrid poplar in temperate regions for use as a feedstock for heat and power applications [10] as well as cellulosic biofuels [11, 12].

Hybrid poplars have been proposed as an ideal woody feedstock for cellulosic biofuels due to a number of factors that include short generation time and rapid growth rate, ease of propagation through vegetative propagation and regrowth following harvest, and substantial genetic diversity and tractability [9]. Field trials with hybrid poplar plantations using cultivation strategies that include single-stem production or short-rotation coppicing have demonstrated biomass yields in the range of 4.5–13.5 dry Mg/ha/year for Wisconsin, Michigan, and Minnesota [10, 13].

*Eucalyptus* ssp. are high-yielding, high-bulk density feedstocks for fiber and potentially suitable as a feedstocks for bioenergy in tropical and subtropical regions. Eucalypts are the most widely cultivated commercial hardwood globally with over 20 million ha in cultivation [14] with the majority of Brazilian eucalypts grown on 5- to 10-year rotations [15]. Furthermore, these trees have demonstrated biomass productivities in the range of 19–31 dry Mg/ha/year in Australia, Florida, and Brazil [9, 15]. Additionally, freeze-tolerant eucalyptus varieties have recently been engineered and have been proposed to offer enormous potential for utilization in plantation forestry in the southeastern U.S. if regulatory hurdles and public opinion concerns can be overcome [14, 16].

While woody biomass exhibits many positive features as outlined above, the higher recalcitrance of these feedstocks to deconstruction relative to herbaceous biomass presents additional challenges. Pretreatment approaches and conditions that are optimal for herbaceous feedstocks may be ineffective for select woody feedstocks, necessitating either substantially harsher pretreatment conditions or potentially new pretreatment strategies. The successful integration of pretreatment technologies with ethanol fermentation relies on careful consideration of chemical inputs to the pretreatment and their interactions with fermentative microbes. As one example, solvent recovery is critical in pretreatments employing solvents and/or reagents other than water such as co-solvent, ionic liquid, and ammonia-based pretreatments. In addition, pretreatments often generate compounds from the biomass that act as inhibitors of enzymatic hydrolysis [17] and fermentation [18]. As examples, pretreatments performed under acidic conditions can lead to the formation of furans from the dehydration of sugars; mild alkaline oxidative pretreatments could result in formation of phenolic acids; and in the case of ionic liquid or organosolv pretreatments, the solvent itself may inhibit enzymes and could be toxic to fermentative microbes if not removed [18]. Some pretreatments are capable of fractionating biomass through the action of the solvent preferentially extracting and solubilizing specific categories of cell wall biopolymers (i.e., hemicelluloses and/or lignin and their degradation products) to yield process streams enriched or depleted in these biomass fractions.

Making comparisons and drawing conclusions between individual pretreatment studies can be problematic due to a number of contributing factors. As one example, feedstock variability, even when utilizing the same plant species, can contribute to differences in pretreatment results as biomass feedstocks can exhibit substantial differences in properties that include differences in genotype, growth and harvest conditions, particle size, and storage history. Standardized assessment of pretreatment efficacy by enzyme hydrolysis is another challenge with potential sources of variability including differences in enzyme source, batch-to-batch variability between commercial cocktails, loss of enzyme activity with age, differences in the approach utilized to assay enzyme activity/protein content to determine enzyme loading, and potentially other minor differences in analytical protocols. As such, standardized benchmarking of different biomass deconstruction and conversion approaches is important for assessing process performance. Single laboratory comparisons of different pretreatments on single feedstocks have been performed on feedstocks that include a hardwood [19], a softwood [20], or sugarcane bagasse [21]. A number of larger scale multi-laboratory comparative studies for benchmarking pretreatments have been performed through the Biomass Refining Consortium for Applied Fundamentals and Innovation (CAFI) for corn stover [22, 23], hybrid poplar [12], and switchgrass [24, 25], while later work compared pretreatment technologies studied within the U.S. DOE’s Bioenergy Research Centers (BRCs) for corn stover [25–27].

Building on these previous studies, the goal of the work presented here was to compare the performance of three pretreatments: (1) an acidic solvolysis pretreatment employing THF and water co-solvents (co-solvent-enhanced lignocellulosic fractionation, CELF) [28–32] (2) a high-solid loading pretreatment with the ionic liquid cholinium lysinate ([Ch][Lys]) that has the potential to be derived from lignocellulosic biomass [33–37], and (3) two-stage Cu-catalyzed alkaline hydrogen peroxide pretreatment (Cu-AHP) utilizing an alkaline pre-extraction followed by an Cu-catalyzed alkaline–oxidative stage [38–42]. The efficacy of these three pretreatments was evaluated on two different hardwood feedstocks, a hybrid poplar and a eucalyptus, and the impact of pretreatment on hydrolysis yields and lignin properties, including the lignin’s susceptibility to depolymerization, was assessed. The first component of this work was to evaluate the susceptibility of the pretreated biomass to hydrolysis by cellulolytic enzymes, and this reactivity was related to both structural and compositional changes to plant cell wall as a consequence of pretreatment. The second component was to determine comprehensive mass balances on the pretreatments. This included assessing the solubilization, depolymerization, and conversion of cell wall biopolymers. An important feature of the pretreatments in this study was that all three are capable of yielding lignin-enriched process streams, with preliminary evidence demonstrating that these lignins may exhibit properties amenable to further valorization. As the final component of this work, we characterized the yields, structural properties, and susceptibility of the pretreatment-soluble and insoluble lignin fractions generated by these pretreatments for depolymerization to aromatic monomers.

## Results and discussion

### Compositional changes and mass solubilization

**Fig. 1:**
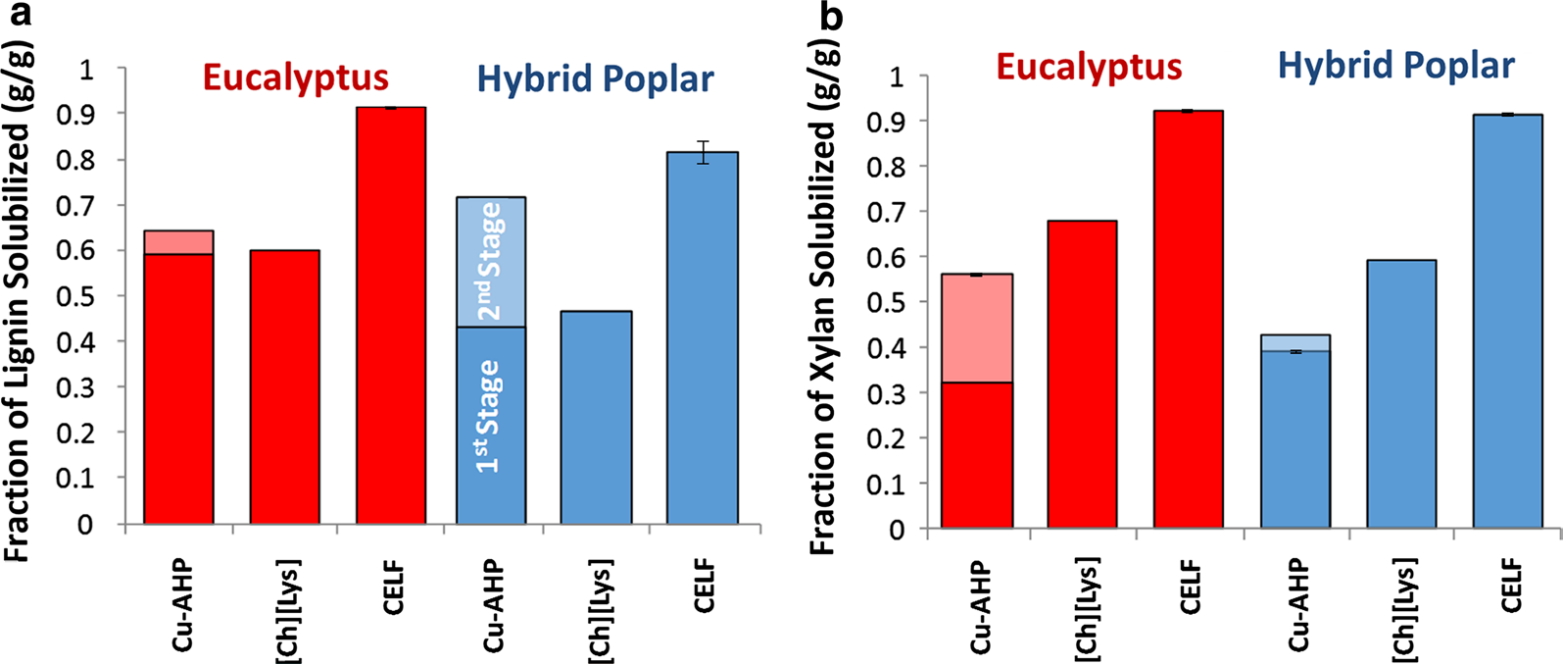
Solubilization of **a** lignin and **b** xylan during each of the pretreatments as determined by a combination of composition analysis and mass yield following pretreatment. For Cu-AHP, the solid color represents the first stage of the pretreatment (alkaline pre-extraction), while the semitransparent color represents the second stage (alkaline oxidative Cu-AHP delignification) with all values on a per mass original biomass basis

One of the primary outcomes of chemical pretreatments is the solubilization, redistribution, chemical modification and/or reordering of the biopolymers within plant cell walls, the extent of which depends on the pretreatment chemistry and conditions [43]. These changes result in differences in cell wall bulk composition as well as differences in other properties including cell wall polysaccharide accessibility to cellulolytic enzymes. The three pretreatments compared in this work were alkaline pre-extraction followed by Cu-catalyzed oxidative delignification (two-stage Cu-AHP), an acidic organosolv pretreatment using THF (CELF pretreatment), and an ionic liquid pretreatment ([Ch][Lys]). The pretreatment conditions were not necessarily optimized for the feedstocks, however, and preliminary screening suggested that a more “severe” pretreatment on the eucalyptus would improve hydrolysis yields. Consequently, for eucalyptus, a higher temperature was used during the 1st stage (i.e., the alkaline pre-extraction) of Cu-AHP pretreatment (150 °C vs. 120 °C), while a longer time was used for the CELF pretreatment (25 vs. 15 min) to improve the delignification and hydrolysis yields [28, 44]. All pretreatments resulted in significant changes in bulk composition of the biomass (Additional file 1: Table S1). The changes are due to solubilization and removal of lignin and xylan, and significant differences in the extent of lignin and xylan removal are apparent for the three pretreatments (Fig. 1). It can be observed that all three pretreatments removed a significant amount of the xylan and lignin with the general trends of CELF > [Ch][Lys] > Cu-AHP for the xylan for both feedstocks, while the trend for lignin can be observed to be CELF > Cu-AHP > [Ch][Lys]. Lignin and xylan removal during alkaline pretreatments at low temperature can be considered to be primarily a consequence of solubility effects rather than significant covalent modification [45], while xylan removal during the acidic THF pretreatment may be attributed to both solvation of polymeric xylan and its subsequent depolymerization to shorter oligomers and monomers [46].

### Enzymatic hydrolysis yields

**Fig. 2:**
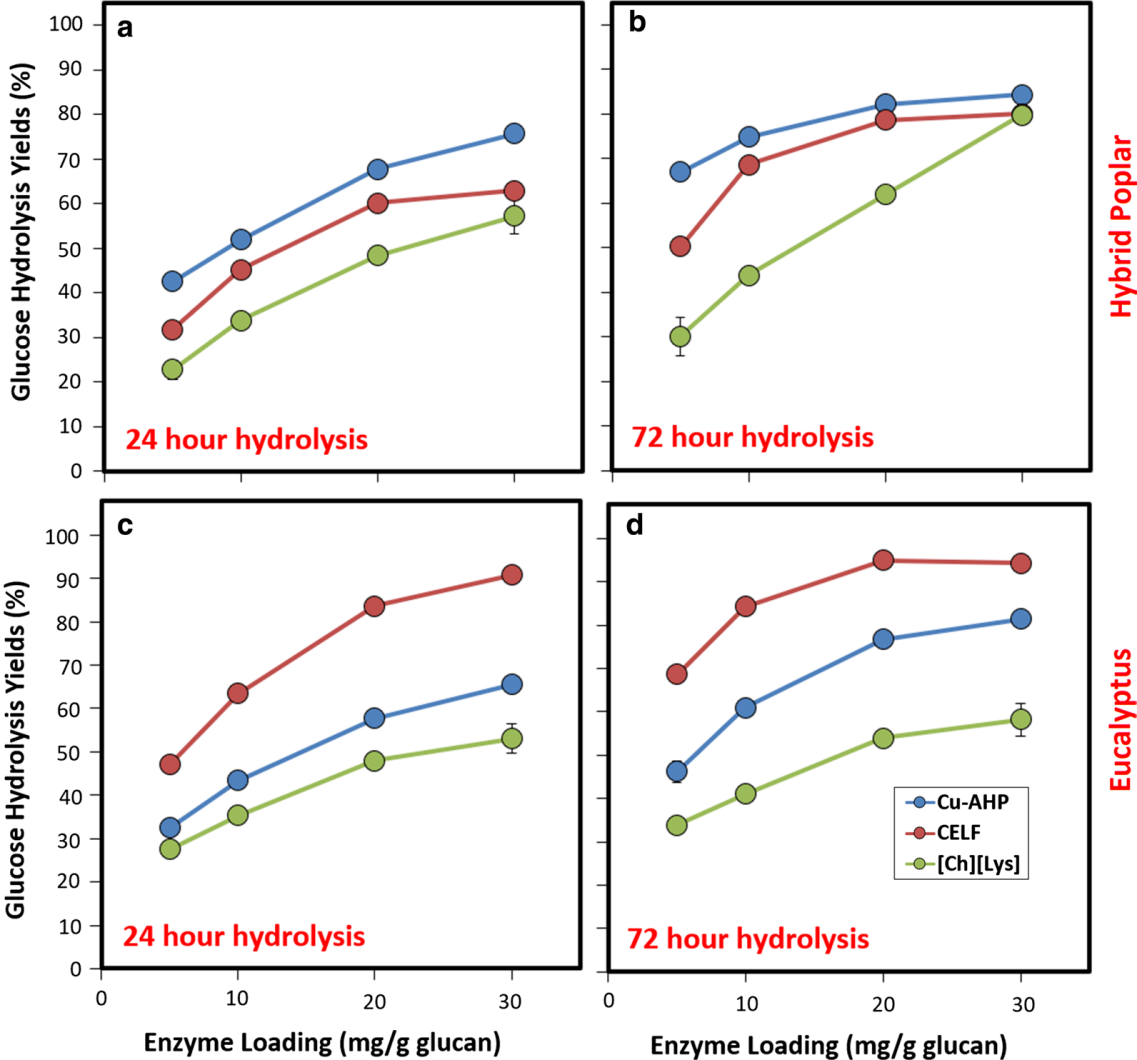
Enzymatic hydrolysis glucose yields for pretreated solids of hybrid poplar (**a** and **b**) and eucalyptus (**c** and **d**) prepared by Cu-AHP, CELF, and [Ch][Lys] pretreatments as a function of enzyme loading (mg protein/g glucan in pretreated solids) and hydrolysis time. Enzymatic hydrolysis was performed at a 10% (wt/vol) solids loading with the pH buffered at 5.0 for 24 or 72 h

Enzymatic hydrolysis yields for glucose (Fig. 2) and xylose (Additional file 1: Figure S1) were determined as a function of enzyme loading. It should be noted that hydrolysis yields are calculated based on original glucan or xylan in the biomass; so, any structural polysaccharides lost during pretreatment contribute to decreased yields for enzymatic hydrolysis. The results for glucose hydrolysis yields demonstrate significant differences between pretreatments as a function of enzyme loading (Fig. 2). Several key observations can be made from these results. One obvious trend is that, as expected, the hydrolysis yields increase with increasing enzyme loading for all pretreatments and that the glucose yields at 72 h for both feedstocks appear to approach their asymptotic maximum values for the Cu-AHP- and CELF-pretreated biomass. For hybrid poplar, glucose yields exceeding 80% of theoretical were observed for all three pretreatments (Fig. 2b) at the highest enzyme loading (30 mg/g) and longest incubation time (72 h). For both feedstocks, the [Ch][Lys]-pretreated biomass consistently exhibited lower hydrolysis yields. One expected reason for this is the inhibitory effect of pretreatment-derived compounds on the enzymatic hydrolysis. Specifically, it should be considered that CELF, being primarily a fractionating treatment, removes the biomass of pretreatment-derived solubles and solvents during the isolation of the solids for enzymatic hydrolysis. The implications of this are that the only factors limiting hydrolysis yields in the CELF-pretreated biomass are intrinsically derived from cell wall structural contributions such as polysaccharide accessibility and cell wall porosity (i.e., biomass recalcitrance) rather than pretreatment-derived solubles. The [Ch][Lys]-pretreated biomass, on the other hand, had substantially more pretreatment-derived solubles present during enzymatic hydrolysis, and these solubles are a likely contributor to the lower observed hydrolysis yields [47]. The Cu-AHP-pretreated biomass only contained solubles derived from the degradation of plant cell wall biopolymers and extractives solubilized during the second pretreatment stage, which are anticipated to contribute only minimally to the inhibition of enzymatic hydrolysis.

With respect to the values for the hydrolysis yields, the pretreated hybrid poplar is slightly less recalcitrant than the eucalyptus when comparing hydrolysis yields for the Cu-AHP and [Ch][Lys] pretreatments. For the Cu-AHP, this is manifested as slightly lower yields for the eucalyptus at low enzyme loadings and shorter hydrolysis times, although the maximum glucose yields (~ 80%) are comparable at the highest enzyme loadings (30 mg/g) and incubation times (72 h). A likely contributor to this higher recalcitrance in the eucalyptus is the higher lignin content (30% by mass) relative to the poplar (24% by mass) as well as potentially the higher extractives content, which are known to inhibit cellulase activity [48] and decrease the efficacy of pretreatment and enzymatic hydrolysis [49]. When similar conditions are compared for the [Ch][Lys] pretreatment, the poplar gives higher yields for most conditions, presumably due to a combination of the lower intrinsic recalcitrance of the biomass as well as the (unquantified) pretreatment-solubilized inhibitors of hydrolysis. For the case of the CELF pretreatment, exceptionally high glucose yields could be obtained for the eucalyptus (i.e., > 95%), although it should be considered that slightly more severe pretreatment conditions were employed for the eucalyptus (25 min at 160 °C) versus the poplar (15 min at 160 °C). When comparing the xylose hydrolysis yields (Additional file 1: Figure S1), it can be observed that for all pretreatments, the xylose yields were low with maximum values ranging from 8% (CELF) to 51% (Cu-AHP) for the poplar and 8% (CELF) to 32% (Cu-AHP) for the eucalyptus. The reason for these low yields is that a significant fraction of the xylan was solubilized during the pretreatment step and was not available for enzymatic conversion. This solubilization does not necessarily represent a loss, however, as pretreatment streams rich in solubilized xylan, xylose, and degradation products could theoretically be utilized elsewhere in the process.

### Cell wall properties contributing to biomass recalcitrance and graphical mass balances

**Fig. 3:**
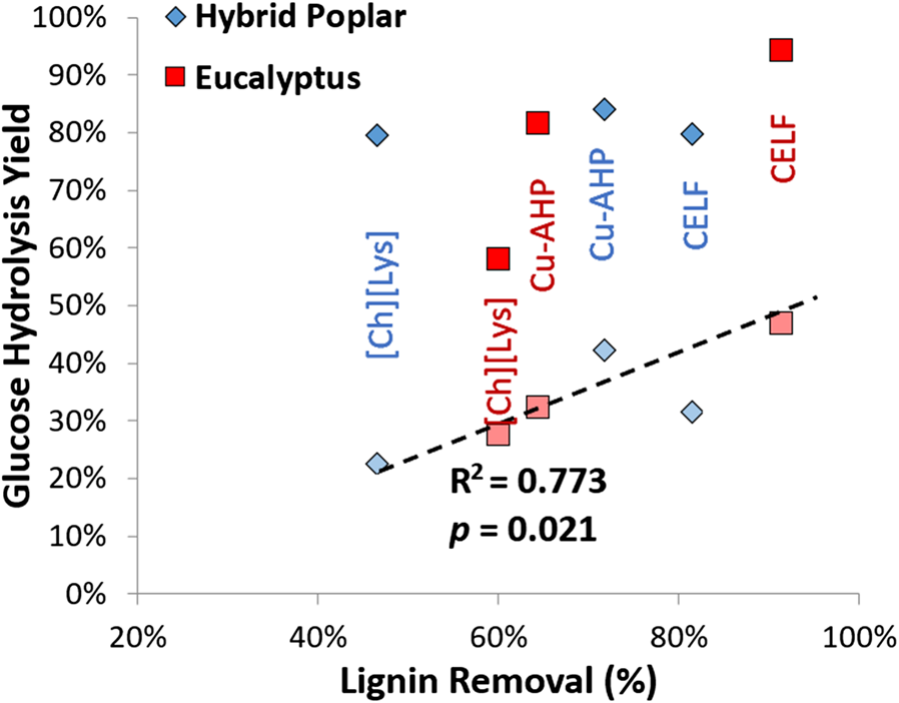
Correlating enzymatic hydrolysis glucose yields with lignin removal. Solid data points represent 72-h hydrolysis yields at an enzyme loading of 30 mg/g glucan, while semi-transparent data points represent 24-h hydrolysis yields at an enzyme loading of 5 mg/g glucan

All three pretreatments studied in this work are delignifying pretreatments and resulted in significant changes in cell wall composition as demonstrated in Fig. 1. As cell wall lignin content is one of the primary contributors to cell wall recalcitrance, plotting lignin removal versus glucose hydrolysis yields can provide insight into how much of a role lignin removal may play in determining hydrolysis yields. As shown in Fig. 3, plotting hydrolysis yields versus lignin removal for both feedstocks at low-yield conditions (5 mg/g enzyme loading, 24-h hydrolysis) results in positive linear correlations. Notably, a strong, positive linear correlation (*R*^2^ = 0.773; *p* value = 0.021) can be observed for the low enzyme loading, demonstrating that lignin removal is a strong predictor of hydrolysis yields. The only outlier is the high-yield condition for the [Ch][Lys]-pretreated eucalyptus. It can be speculated that the lower than expected yield for this condition may be due to the contribution of pretreatment-derived solubles (e.g., the ionic liquid itself, soluble lignin and/or xylan, or extractives). These types of correlations between lignin removal and hydrolysis yields are not unexpected, and have been demonstrated previously for a wide range of feedstocks and pretreatment chemistries, including flow-through dilute acid pretreatment of corn stover [50], two-stage Cu-AHP pretreatment of hybrid poplar [41], alkaline and alkaline–oxidative delignification of hardwoods and softwoods [51], and alkaline hydrogen peroxide delignification of corn stover and switchgrass [52], among others.

**Fig. 4:**
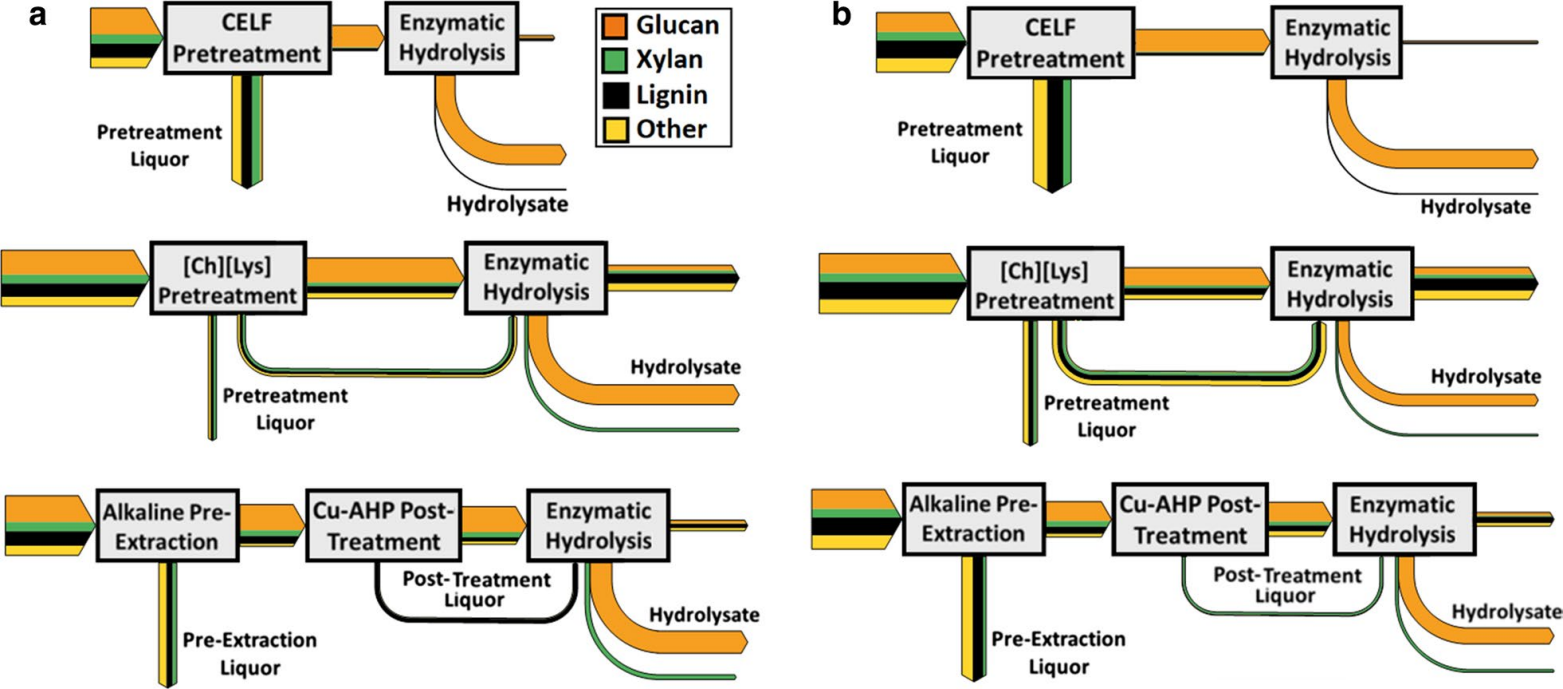
Sankey diagrams representing mass flows through deconstruction for **a** hybrid poplar and **b** eucalyptus. Hydrolysis yields are based on 30 mg/g enzyme loading for 72 h and pretreatment liquor compositions are based on mass differences

Sankey diagrams can be employed as a tool to visualize the flow of mass and energy through conversion processes [53], and in this work, the compositional analysis results are integrated with mass yields and hydrolysis yields to compare mass component flows for the three processes (Fig. 4). The results were calculated using enzymatic hydrolysis yields experimentally determined for 72-h hydrolysis at 30 mg/g glucan enzyme loading. Key observations from these plots were that the vast majority of the lignin and xylan during the CELF pretreatment partition into the solvent phase and are removed during pretreatment (also clear from Fig. 1) and may be available in subsequent conversion or utilization steps. Another key result is that the majority of the pretreatment-solubilized lignin and xylan in the [Ch][Lys] pretreatments continue through the enzymatic hydrolysis step and ultimately ends up in the hydrolysate.

### Impact of pretreatment on recovered lignin properties

All three pretreatments function, at least partially, as delignifying pretreatments and offer the opportunity to fractionate the biomass to yield a lignin stream that may be amenable to valorization. As discussed previously, the lignin structural features/properties (e.g., functional groups, molar mass distributions, solubilities, monomer composition, interunit linkages, etc.) are key determinants in their suitability for a target application. Additionally, these properties are a complex function of the lignin’s biological origin and its processing history. As examples, for utilization of lignin as a phenol replacement in phenol–formaldehyde resin applications, a high content of unsubstituted aromatic sites in a terminal phenolic group is necessary for the lignin to be incorporated into the polymer [54]. For use as a polyol in polyurethane applications, a high content of accessible hydroxyl groups is a key property that sets the value of lignin and can result in increased incorporation into polymer products [55], with aliphatic hydroxyl groups exhibiting preferential reaction with isocyanates than aromatic hydroxyls. As a third example, lignin solubility in organic nonpolar solvents and its contribution to increased viscosity in reaction solvents are important properties for certain polymer applications that include polyurethanes and thermoset resins [56, 57].

Lignin depolymerization to aromatic monomers or low molecular weight oligomers is one route to convert lignin into valuable and renewable intermediate chemicals to improve the economics of biofuels [58]. These depolymerization approaches typically target ether linkages, primarily the β-*O*-4 bond that, when cleaved, produce excised fragments that can then be more easily solubilized by the solvents employed during pretreatment. For efficient depolymerization of lignin to aromatic monomers, necessary features include a high fraction of the monomers linked by ether bonds such as the β-*O*-4 bond as well as minimal pretreatment-induced repolymerization [59]. Notably, it has been demonstrated that acid-catalyzed lignin depolymerization occurs under conditions that may be encountered during acidic pretreatments, and, at high severity conditions, are known to drive certain lignin repolymerization either by condensation reactions through reactive carbocations at the α position [59] or through reactive aldehydes at the β position of the lignin side chains [60]. For CELF pretreatment, THF–water is an excellent “theta” solvent for lignin that, when combined with dilute acid, achieves high lignin depolymerization and solubilization at lower severity conditions than water-only pretreatments [61]. To ensure that lignin fragmentation is dominant over condensation, CELF pretreatment is maintained at or below 160 °C to solubilize lignin, while avoiding the production of undesired lignin condensation products known to form at higher severities [29, 62, 63].

**Fig. 5:**
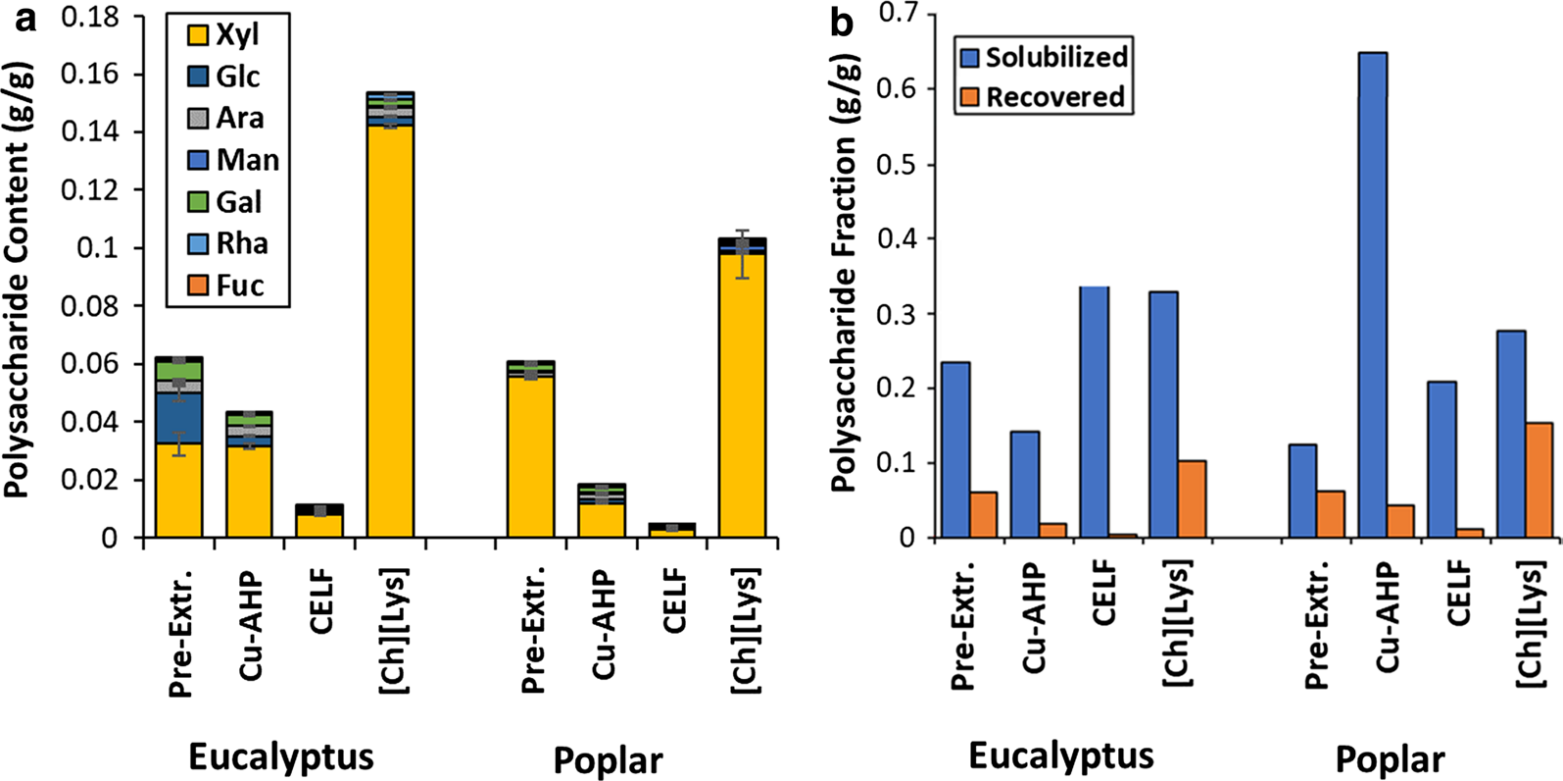
Polysaccharides in lignin-rich precipitates including **a** polysaccharide content and composition and **b** polysaccharide content relative to solubilized polysaccharide mass abundance. “Pre-Extr.” refers to the alkaline pre-extraction step or the first stage of the Cu-AHP process, while “Cu-AHP” refers to the second step or the alkaline oxidative Cu-AHP stage

**Fig. 6:**
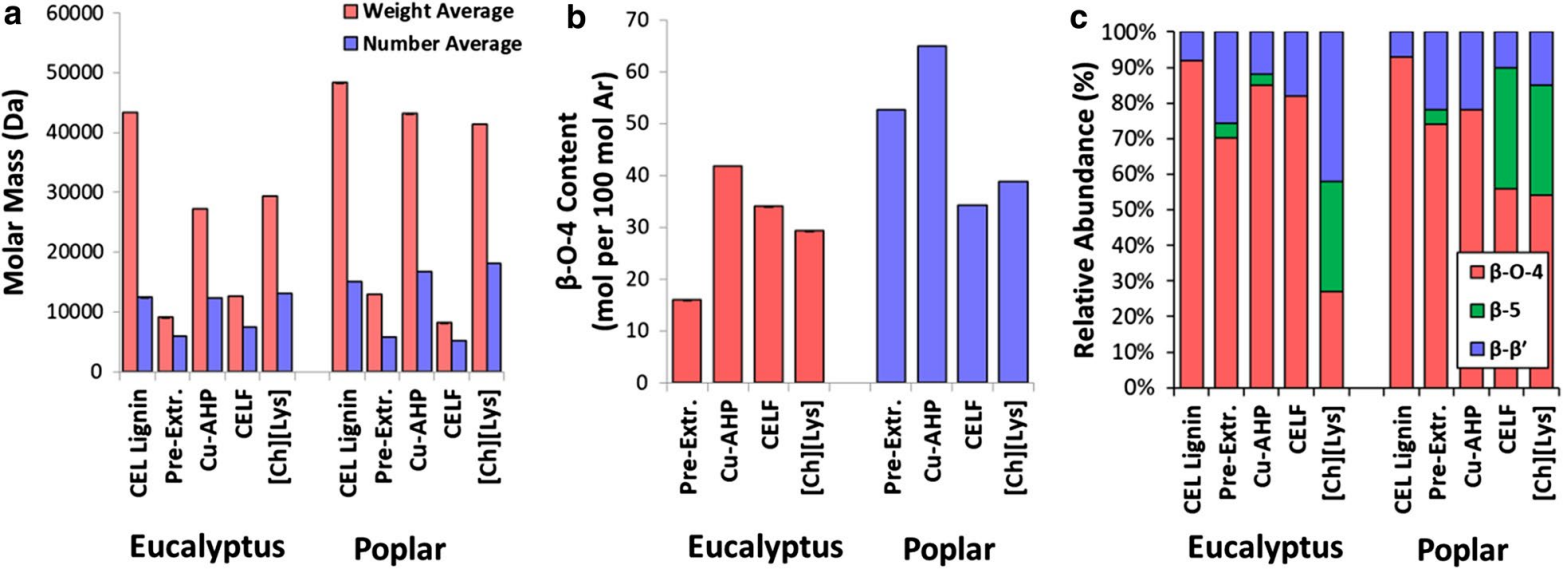
Quantified lignin properties for pretreatment-solubilized lignins and reference “native” cellulolytic lignin (CEL lignin), including **a** SEC-estimated weight and number average molar masses, **b** β-*O*-4 content determined by ^13^C NMR, and **c** HSQC NMR-determined relative abundances of interunit linkages within the lignins. “Pre-Extr.” refers to the alkaline pre-extraction step or the first stage of the Cu-AHP process, while “Cu-AHP” refers to the second step or the alkaline oxidative Cu-AHP stage

In this work, lignins (or lignin-rich precipitates) recovered from the liquid phase for the three pretreatment strategies were subjected to several characterization approaches, and the susceptibility of these lignins to depolymerization by thioacidolysis was assessed. These characterization approaches include structural polysaccharide content of the recovered lignin-rich precipitates (Fig. 5a), determination of molar mass distributions by SEC (Fig. 6a), β-*O*-4 content as determined by quantitative ^13^C NMR (Fig. 6b), and non-quantitative relative abundance of interunit lignin linkages as assessed by HSQC NMR (Fig. 6c). For the cell wall-derived structural polysaccharides co-precipitating with the lignin (Fig. 5a), it can be observed that both the polysaccharide abundance and distribution vary depending on the feedstock and pretreatment. Specifically, xylan is the most abundant polysaccharide and comprises from 52% of the polysaccharide content (Cu-AHP pre-extraction for eucalyptus) to more than 90% (CELF for both feedstocks and Cu-AHP pre-extraction for poplar). For the recovered polysaccharide abundance, the clear trend for both feedstocks is [Ch][Lys] > 1st-stage Cu-AHP > 2nd-stage Cu-AHP > CELF. The low polysaccharide content of the CELF lignins (1.0% and 0.4% by mass for poplar and eucalyptus, respectively) is hypothesized to be due to two contributing factors. The first is that the CELF pretreatment is performed under acidic conditions such that the majority of the solubilized xylan is hydrolyzed to xylose [28, 32], thereby resulting in minimal soluble xylan oligomers that are available to co-precipitate with the lignin. The second factor is that lignin precipitation by water dilution or by boiling off the THF also results in partitioning of the sugar monomers and low-MW oligomers into the aqueous phase rather than precipitation with the lignin [62]. At the other extreme, [Ch][Lys] contains from 10.3% (poplar) to 15.3% (eucalyptus) polysaccharides in the recovered lignins. However, for all pretreatments, when the hemicellulose solubilized during the pretreatment process is compared to the hemicellulose recovered in the precipitate (Fig. 5b), it can be observed that the relative abundance of the hemicellulose is significantly lower in the recovered precipitates than what is solubilized. For the CELF and [Ch][Lys] cases, this may be attributed to the partial or complete conversion of the solubilized hemicelluloses into other water-soluble products. For all three cases, another explanation for the discrepancy is that a fraction of the hemicelluloses is generally less amenable to precipitation under the conditions used relative to the pretreatment-solubilized lignins. For example, solubilized xylan may comprise multiple populations of polymers exhibiting differences in molar mass and degree of substitution that has in the past been linked to both its solubility [64] and its degree of association with cellulose [65].

Recovered biopolymers fractionated during the pretreatments were next assessed for molar mass distributions by SEC (Fig. 6a, see Additional file 1: Figure S2 for the elution profiles). From these results, significant differences in the apparent molar masses of the recovered biopolymers can be observed. Importantly, it is well established that while trends or qualitative differences between lignin samples may be observed, quantitative values for SEC/GPC-determined molar masses of lignins suffer from a lack of exactness when results are compared between methods due, at least in part, to lignin’s tendency for self-aggregation [66]. This data set includes characterization of a relatively “native” reference lignin (CEL lignin) that was recovered by a combination of ball milling, cellulose decrystallization, and enzymatic hydrolysis. From these results, it can be observed that the “native” lignin, the Cu-AHP (2nd-stage) lignin, and the [Ch][Lys] lignin exhibited the highest number average ($\bar{M}_{\text{N}}$) and weight average ($\bar{M}_{\text{W}}$) molar masses, while the 1st-stage Cu-AHP (pre-extraction) and CELF pretreatments yielded recovered biopolymers with significantly lower values for these properties (Fig. 6a). For the lignins recovered from the CELF pretreatments, the molar masses are consistently low for both feedstocks ($\bar{M}_{\text{N}}$ of 7.5 and 5.2 kDa for eucalyptus and poplar, respectively). This is likely due to fragmentation during CELF pretreatment that may result in more acid-catalyzed cleavage of β-*O*-4 bonds with potentially low levels of condensation as reported in prior work [62]. The molar masses of the lignins recovered from the [Ch][Lys] pretreatments were consistently high ($\bar{M}_{\text{N}}$ = 13.2 and 18.1 kDa for eucalyptus and poplar, respectively). One potential reason for these higher observed molar masses may be due to the higher content of contaminating polysaccharides (10.3–15.5% by mass from Fig. 5a) that may skew the results towards higher molar masses. Using a range of characterization approaches, native hardwood glucuronoxylans have been estimated to have degrees of polymerization in the range of 150–200 monomer units (corresponding to ~ 22.5–30 kDa) [67, 68]. Thus, the differences in the SEC results for the [Ch][Lys] lignin cannot unambiguously be ascribed to differences in the lignin properties alone.

Next, the β-*O*-4 content of the recovered lignin-rich samples is determined by quantitative ^13^C NMR (Fig. 6b) and the relative abundance of intra-unit linkages is determined using semi-quantitative HSQC NMR (Fig. 6c, see Additional file 1: Figure S4 for complete NMR results and peak assignment and quantification). These results show that both the absolute β-*O*-4 content (Fig. 6b) and the relative β-*O*-4 content (Fig. 6c) exhibit nearly identical trends between pretreatments for both characterization methods. Of the three bond types characterized, the relative abundances of the β-*O*-4 in the “native” lignin are the highest (Fig. 6c), while the relative abundance of the other two types of linkages increases following pretreatment. Notably, it is understood that a β–β linkage is generated during initial monolignol coupling reactions during lignin biosynthesis [69], and as these are not formed during the pretreatment, an increase in the relative abundance of a β–β linkage would indicate a decrease in the β-*O*-4 abundance. The Cu-AHP pre-extraction lignin from eucalyptus exhibits much lower β-*O*-4 content relative to the 2nd-stage eucalyptus Cu-AHP lignin (Fig. 6b) or either of the poplar lignins from the Cu-AHP process. A substantially higher temperature was utilized for the eucalyptus pre-extraction (150 °C) relative to the temperature used for the poplar (120 °C) due to the higher recalcitrance of the eucalyptus. Our hypothesis is that this increase in temperature not only solubilized more lignin from the eucalyptus during the 1st-stage Cu-AHP (Fig. 1), but also it presumably resulted in more cleavage of β-*O*-4 bonds in the pretreatment-solubilized lignin. This agrees with the results for molar mass (Fig. 6a) which show that the eucalyptus Cu-AHP pre-extraction exhibits one of the lowest number average molar masses.

### Lignin depolymerization and correlation of lignin properties with aromatic monomer yields

**Fig. 7:**
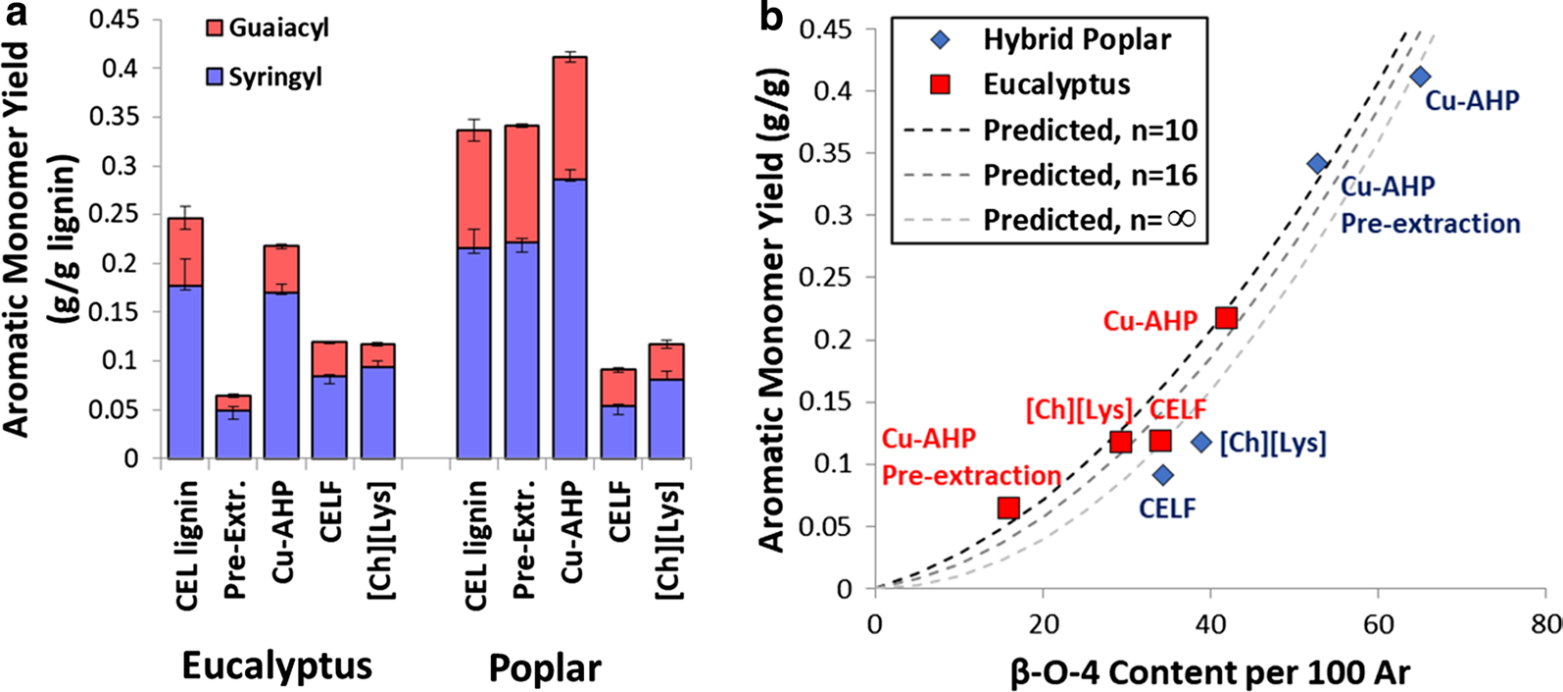
Results for **a** phenolic monomer yields from quantitative thioacidolysis and **b** correlation between aromatic monomer yield by quantitative thioacidolysis and β-*O*-4 content of the pretreatment-solubilized and recovered lignin as determined by ^13^C NMR. “Pre-Extr.” refers to the alkaline pre-extraction step or the first stage of the Cu-AHP process, while “Cu-AHP” refers to the second step of the alkaline oxidative Cu-AHP stage. “CEL” refers to the cellulolytic lignin used as a control as “native” lignin

The fractionated lignins were next subjected to thioacidolysis as a characteristic depolymerization targeting the β-*O*-4 bond within the lignin polymer. From this analysis, both monomer yields and the syringyl to guaiacyl (S/G) ratios were determined. First, the S/G ratios determined by thioacidolysis were compared to those obtained by HSQC NMR with the results plotted in Additional file 1: Figure S5. While exhibiting a different range of absolute numbers, the correlation between the two approaches yields an *R*^2^ value of 0.88 with a *p* value of 0.0028. While the S/G ratios are determined on a mol/mol basis, the results for depolymerization (Fig. 7a) are determined on a “per mass lignin” basis, where the lignin in the denominator is the non-polysaccharide content of the recovered biopolymer sample. The mass of a “monomer” in the numerator is corrected as the expected mass of a unit within lignin (syringyl monomer: 226 g/mol; guaiacyl monomer: 196 g/mol), so that these yields can be subsequently correlated with predicted yields. The native lignin is intended to demonstrate the approximate upper limit for monomer yields as these are expected to retain the majority of the β-*O*-4 bonds while not exhibiting any repolymerization that may occur in the process-modified lignins. From these results, clear differences between feedstocks and pretreatments can be observed, with the “native” lignin and the Cu-AHP (2^nd^-stage) lignin for both feedstocks and the Cu-AHP pre-extraction (1st stage) for the poplar exhibiting the highest values for β-*O*-4 content with 21.8–24.7% aromatic monomer yields for eucalyptus and 33.7–41.2% for the poplar (Fig. 7). The other pretreatment–feedstock combinations resulted in much lower monomer yields with 6.4–11.9% for eucalyptus and 9.1–11.8% for poplar. The lower yield from the Cu-AHP pre-extraction of eucalyptus is hypothesized to be due to the higher temperatures used during the pre-extraction relative to the poplar, consistent with the β-*O*-4 content results. For the ionic liquid and CELF pretreatments, the monomer yields are also comparably low for both feedstocks (9.0–11.8%), indicating significant cleavage of β-*O*-4 during the pretreatment and/or potential repolymerization during the pretreatment.

A second-order functionality between β-*O*-4 content and aromatic monomer yield from lignins have been proposed in the past [70, 71]. Our previous work with fractionated lignins derived from the soda pulping of hybrid poplar demonstrated that the β-*O*-4 content as determined quantitatively by ^13^C NMR as well as GPC-determined molar masses exhibited strong positive correlations with the monomer yields obtained following thioacidolysis [71]. As thioacidolysis targets aryl ether bonds within lignin, this correlation between β-*O*-4 content and monomer yields should be expected. Furthermore, we developed and validated a methodology to predict the maximum theoretical monomer yield based on the probability that a monomer contains two adjacent β-aryl ether bonds or that a monomer at an end of a lignin polymer contains a β-aryl ether bond [56]. This relationship is described by:

1 $$ {\text{Monomer}}\;{\text{Yield}} = \frac{{\left( {n - 2} \right) \cdot \left( {\upbeta\hbox{-}O\hbox{-}4\;{\text{Content}}} \right)^{2} }}{n} + \frac{{2 \cdot \left[ {\upbeta\hbox{-}O\hbox{-}4\;{\text{Content}}} \right]}}{n}, $$

where *n* is the number of aromatic monomers in a typical lignin polymer, β-*O*-4 *Content* is the β-*O*-4 content as a fraction of total linkages, and *Monomer Yield* is the moles of monomer per mole of monomers within the polymer. This approach assumes linear polymers with no crosslinking and the predictive power may be expected to break down for highly process-modified lignins. The results of this model prediction using values of *n* ranging from 10 to ∞ are presented in Fig. 7b alongside the experimental results. While SEC results might suggest degrees of lignin polymerization in the range of 30–50, these values are probably inflated due the quantification method, and actual values are likely to be one half to one quarter of these values [72, 73]. Comparing the model prediction with the experimental monomer yields indicates that this model provides relatively good prediction of monomer yields (Fig. 7b), exhibiting an *R*^2^ value for predicted versus measured of 0.92 for all values of *n* ranging from 10 to ∞. Overall, this provides additional validation of this model while suggesting that pretreatment approaches that preserve β-*O*-4 content (or alternatively integrate lignin depolymerization with pretreatment) are a favored approach if aromatic monomer production is targeted.

## Conclusions

Three diverse pretreatments capable of biomass fractionation were the subject of parallel comparisons on a hybrid poplar and eucalyptus for their impact on cell wall polymer solubilization, enzymatic hydrolysis yields, and lignin properties. It was demonstrated that all three pretreatments were capable of solubilizing a significant fraction of the lignin and xylan and that all pretreatments were capable of achieving high (~ 80%) hydrolysis yields for the hybrid poplar. The eucalyptus was more recalcitrant and resulted in lower hydrolysis yields, with substantially lower yields for the [Ch][Lys] pretreatment, presumably due to the higher lignin content of the eucalyptus and the fact that a substantial fraction of the pretreatment-derived inhibitors in [Ch][Lys] pretreatment is carried forward into the enzymatic hydrolysis stage. The removal of lignin during pretreatment was shown to be a strong predictor of enzymatic hydrolysis yields for low enzyme loadings for both feedstocks for all three pretreatments. The recovered lignins from the pretreatment liquors in each of the pretreatments were characterized and exhibited substantial differences in properties. Namely, the lignins recovered from the [Ch][Lys] pretreatment had a high polysaccharide content (10–15%), while the CELF lignins did not, presumably due to substantial hydrolysis of the solubilized xylan. The lignins exhibited significant differences between samples in both the ^13^C NMR-determined β-*O*-4 content and aromatic monomer yields when subjected to depolymerization by thioacidolysis. The aromatic monomer yields demonstrated second-order functionality with respect to the β-*O*-4 content, suggesting the use of feedstocks with high β-*O*-4 content lignins and pretreatments that preserve these linkages be used if subsequent lignin depolymerization is a goal.

## Methods

### Biomass

The hybrid poplar, *Populus nigra* L. var. charkoviensis × caudina cv. NE-19, was obtained from 18-year-old trees grown at the University of Wisconsin Arlington Agricultural Experiment Station (Arlington, WI) and harvested in 2011 and is identical to the feedstock used in our prior work [39, 41]. Hybrid poplar logs were initially debarked and chipped prior to subsequent milling. The eucalyptus (*Eucalyptus cinerea*) is identical to the feedstock used in prior work by the authors [74] and was originally provided by Idaho National Laboratory. Both biomass feedstocks were subjected to particle size reduction using a Christy-Turner lab mill (Christy-Turner LTD, Ipswich, Suffolk, UK) to pass a 2-mm screen and air-dried to ~ 5% moisture.

### Biomass composition analysis

Prior to and following pretreatment, biomass compositions were determined according to NREL/TP 510-42618 [75] with structural carbohydrates and acetate determined by HPLC (Agilent 1200 Series) using an Aminex HPX-87H column (Bio-Rad, Hercules, CA, USA). The chromatography was performed at 65 °C with a mobile phase of 5.0-mM aqueous H_2_SO_4_ at a flowrate of 0.6 mL/min and detection by refractive index. Mass balances were accomplished using a combination of composition prior to and following pretreatment stages and mass loss during pretreatment. Composition following enzymatic hydrolysis was estimated based on glucose and xylose solubilized, while mass lignin and xylan solubilized during each stage was estimated by difference. Mass flows determined from these mass balances were plotted in Sankey diagrams utilizing the e!Sankey software (ifu Hamburg GmbH, Hamburg, Germany).

### Cu‑AHP pretreatment

Alkaline pre-extraction of hybrid poplar and eucalyptus biomass was carried out at 10% solids loading (w/w). Five g (dry basis) of hybrid poplar or eucalyptus was incubated with 200-mg NaOH/g biomass for 1 h in a 100-mL volume capacity Parr 4560 Mini Benchtop reactor with electric heating (Parr Instrument Company, Moline, IL). The reaction conditions for hybrid poplar were 120 °C for 60 min (including 15-min heat-up and 10-min cool-down time) and for eucalyptus, these were 150 °C for 60 min (including 22-min heat-up and 10-min cool-down time). After 1 h of incubation, the remaining insoluble biomass was thoroughly washed with deionized water, air-dried, and subjected to 23 h of Cu-AHP pretreatment. Cu-AHP pretreatment was performed at room temperature at 20% solids loading (w/w) in a flask. Biomass (10 g, dry basis) was incubated with 100-mg NaOH/g biomass, 1-mM copper (as CuSO_4_), and 2-mM bipyridine. Hydrogen peroxide (30% v/v stock solution) was added to the reaction mixture in batches over a 10-h period to a final loading of 100 mg H_2_O_2_/g biomass as described in our prior work [41]. Following the final addition of H_2_O_2_, the mixture was incubated for an additional 13 h (24 h total reaction time). To recover the Cu-AHP lignins, following alkaline pre-extraction or Cu-AHP pretreatment, the liquid phase was separated from the solid phase via filtration and the filtrate was acidified to pH 2.0 with 72% (w/w) sulfuric acid. The precipitate was recovered by filtration, washed with aqueous sulfuric acid (pH 2.0), and finally washed by resuspending in deionized water. The suspension was centrifuged and the liquid phase was decanted and the precipitate was collected and lyophilized for subsequent analysis.

### CELF pretreatment

CELF pretreatment was performed in a Parr reactor heated by a 4-kW fluidized sand bath, as described in previous studies [28, 30]. Pretreated eucalyptus and poplar biomass were prepared at the following reaction conditions: 160 °C, 1:1 THF:water (v/v), and 0.5 wt% sulfuric acid loading based on the total liquid mass. The eucalyptus biomass was treated for 25 min at 12.5% solids whereas poplar was treated for 15 min at 15% solids loading. The remaining solids after completion of the treatment were thoroughly washed with the distilled water. The CELF lignin samples were recovered by precipitation from the pretreatment liquors by dilution with water at 4:1 ratio of water:liquor by volume. The precipitated lignin was then vacuum filtered through a paper filter and washed once with diethyl ether and three times with water. The resulting powder was collected after drying at 45 °C for 2 days.

### [Ch][Lys] pretreatment

The ionic liquid [Ch][Lys] was synthesized as reported previously [34]. The pretreatment was performed using 0.5 g (dry basis) biomass that contained 11.1% moisture for the eucalyptus and 6.1% for the poplar. This biomass was incubated with 0.5 g of [Ch][Lys] at 100 °C for 5 h as described in the prior work [76]. After completion of the incubation, 4.0-g water was added and the pH was adjusted to 5.0 using HCl and, following centrifugation, 2.0 g of liquid phase was removed from the supernatant. Recovery of lignin from the [Ch][Lys] pretreatment liquor for characterization was performed as in prior work [77]. Briefly, the slurry following pretreatment was subjected to 3 cycles of water washing and solid/liquid separation by centrifugation (4000×*g*). The supernatant (pH > 10) was combined from the washes and further filtered using a 0.45 µm membrane. The pH of the liquid fraction was then adjusted to ~ 2 with 6 N HCl to precipitate the lignin. Next, the precipitated lignin was separated by centrifugation and lyophilized.

### Enzymatic hydrolysis

The pretreated biomass mixture from each of the three pretreatments was appropriately diluted to obtain 10% solids loading (wt to liquid wt) for enzymatic hydrolysis. The ionic liquid pretreatment slurry still contained 8% (wt to water wt) [Ch][Lys] in addition to pretreatment-solubilized organics, while the Cu-AHP solids contained all the pretreatment-solubilized organics as well all the pretreatment-derived Na^+^ from the second stage of pretreatment. The slurry was slowly titrated with 72% (w/w) H_2_SO_4_ to adjust the pH to 5.0 prior to the addition of 1 M citric acid buffer (pH 5.0) at a final concentration of 50 mM. An enzyme cocktail consisting of a 1:1 ratio of Cellic CTec3 and HTec3 (Novozymes A/S, Bagsværd, DK) on a protein basis (protein content provided by the manufacturer) at loadings of 5, 10, 20, and 30 mg protein/g glucan in the pretreated solids was added to the hydrolysis reaction. The total aqueous volume of the reaction was then adjusted by the addition of deionized water to achieve the target solids loading. The flasks containing biomass slurry and enzymes were incubated at 50 °C on an orbital shaker at 210 rpm with samples taken for sugar analysis at 24 and 72 h. Following enzymatic hydrolysis, the amount of glucose and xylose released in the supernatant was quantified by high-performance liquid chromatography (HPLC; Agilent 1260 Series equipped with a refractive index detector) using an Aminex HPX-87H column operating at 65 °C, a mobile phase of 5.0-mM aqueous H_2_SO_4_, and a flow rate of 0.6 mL/min. It should be noted that this column does not resolve galactose, mannose, and xylose, and quantified xylose, therefore, includes any released mannose and galactose. Standard curves using glucose and xylose were prepared to calculate the sugar concentrations in the samples. The sugar yields (glucose and xylose) were calculated by dividing the amount of released sugar by the total sugar content of the biomass (as monomer) prior to pretreatment as described in our prior work [17], with the final yields corrected to a “per original glucan” basis which was calculated from a combination of mass loss during pretreatment and change in composition when these data are available.

### Generation of “native” celluloytic lignins

A “native” cellulolytic lignin from both the hybrid poplar and eucalyptus was extracted to use as a benchmark for comparison. This lignin was extracted from biomass according to the procedure of Gu et al. [78]. Briefly, the biomass was ball-milled in a TissueLyser II (Qiagen, Hilden, Germany) for a total of 4 h with cooling by liquid N_2_ between milling stages at 15-min intervals. The ball-milled sample was dissolved in 8% LiCl/DMSO at a concentration of 5% by weight, and then stirred at 25 °C for 48 h followed by stirring at 50 °C for 24 h. The biomass was precipitated by dropwise addition of the sample into water, and the precipitate was repeatedly washed with water. This reconstituted biomass sample was then lyophilized before undergoing enzymatic hydrolysis for 72 h with 20 mg protein per g biomass using CTec2 and HTec2 (Novozymes A/S, Bagsværd, DK) at a 2:1 ratio (protein basis) at 50 °C and pH 5.25 using 0.05 M Na-citrate buffer. Finally, the hydrolyzed solids were separated from the liquid by vacuum filtration and were washed with excess water and lyophilized again.

### Lignin characterization

Quantitative thioacidolysis was performed as described in our previous work [79]. In brief, 2 mg of dried and isolated lignin samples were weighed into glass vials in triplicate and heated with a mixture of dioxane, ethanethiol, and boron trifluoride diethyl etherate to liberate the lignin monomers. The extracted thioether derivatized monomers were subsequently silylated with *N*,*O*-bis-trimethylsilyl-acetamide (BSA) and quantitated using GC–MS analysis (Agilent 7890A/5975C MS). Monomer standards were obtained from the laboratory of Dr. John Ralph (University of Wisconsin, Madison).

The neutral polysaccharide content of the recovered lignins was quantified by GC–MS as the monosaccharide alditol acetates following polysaccharide hydrolysis catalyzed by trifluoroacetic acid as outlined by Foster et al. [80].

Size-exclusion chromatography (SEC) was performed as described in our prior work [41] using an Agilent 1260 series HPLC equipped with a Waters Ultrahydrogel™ 250 (Milford, MA, USA) column and employing a mobile phase of 80:20 (v/v) mixture of 0.1 M NaNO_3_:5.0 mM NaOH/CH_3_CN at a flow rate of 0.6 mL/min at 45 °C and detection by refractive index. Monodisperse polyethylene glycol (PEG) standards were utilized to estimate molar masses, and both number average ($\bar{M}_{\text{N}}$) and weight average ($\bar{M}_{\text{W}}$) molar mass were determined numerically using the “direct standard calibration” method outlined in the literature [81].

For ^13^C NMR, a sample of lignin (120 mg) was dissolved in 600-μL DMSO-*d*_6_. A small amount (2 mg) of the relaxation reagent chromium(III) acetylacetonate was added to the sample. Sonication was used to facilitate dissolution. ^13^C NMR spectra were acquired on a 500-MHz NMR spectrometer (Varian Inova) equipped with a double-resonance broadband probe as outlined in our prior work [71]. Proton decoupling was applied only during acquisition period, i.e., decoupling-NOE. The spectra were acquired from − 15 to 235 ppm with a 90° pulse, a recycle delay of 1.7 s, and an acquisition time of 1.2 s. A total of 10,000 scans were collected. Peak assignments were based on previous literature [82, 83]. For HSQC NMR, the lignin samples were ball-milled and approximately 30 mg were placed in NMR tubes with 600 μL DMSO-*d*_6_. The samples were sealed and sonicated to homogeneity in a Branson 2510 table-top cleaner (Branson Ultrasonic Corporation, Danbury, CT). The temperature of the bath was closely monitored and maintained below 55 °C. HSQC spectra were acquired at 25 °C using a Bruker Avance-600 MHz instrument equipped with a 5-mm inverse gradient ^1^H/^13^C cryoprobe using the “hsqcetgpsisp2.2” pulse program (ns = 200, ds = 16, number of increments = 256, d1 = 1.0 s). Chemical shifts were referenced to the central DMSO peak (*δ*_C_/*δ*_H_ 39.5/2.5 ppm). Peak assignments were made according to the published literature [84].

## Supplementary information

**Additional file 1: Table S1.** Composition and mass yields of hybrid poplar and eucalyptus prior to and following pretreatment. Cu-AHP: Cu-catalyzed alkaline hydrogen peroxide pretreatment; [Ch][Lys]: cholinium lysinate pretreatment; CELF: co-solvent enhanced lignocellulosic fractionation. **Table S2.** Assignments of signals observed in ^13^C/^1^H 2D (HSQC) NMR spectra of lignins. **Figure S1.** Enzymatic hydrolysis xylose yields for pretreated solids of hybrid poplar (**A** and **B**) and eucalyptus (**C** and **D**) prepared by Cu-AHP, CELF, and [Ch][Lys] pretreatments as a function of enzyme loading (mg protein/g glucan in pretreated solids) and hydrolysis time. Hydrolysis was performed at a 10% (wt/vol) solids loading with the pH buffered at 5.0 for 24 or 72 h. **Figure S2.** Size-exclusion chromatography (SEC) elution profiles and estimated values for number average molar mass ($\bar{M}_{\text{N}}$), weight average molar mass ($\bar{M}_{\text{W}}$), and polydispersity index (PDI) for (**A**) eucalyptus and (**B**) hybrid poplar. SEC was performed using a Waters Ultrahydrogel^TM^ 250 column with a mobile phase comprising a 80:20 (v/v) solution of 0.1 M NaNO_3_:0.005 M NaOH/CH_3_CN. **Figure S3.** Summary of the fate of the (A, D) glucan, (B, E) xylan, and (C, F) lignin for the three pretreatments for (A, B, C) hybrid poplar and (D, E, F) eucalyptus. Hydrolysis yields are for 30 mg/g glucan enzyme loading for 72 h. **Figure S4.** ^13^C/^1^H 2D (HSQC) NMR spectra of (a) native cellulolytic eucalyptus lignin, (b) eucalyptus 1st-stage Cu-AHP lignin, (c) eucalyptus 2nd-stage Cu-AHP lignin, (d) eucalyptus CELF lignin, (e) eucalyptus [Ch][Lys] lignin, (f) native cellulolytic poplar lignin, (g) poplar 1st-stage Cu-AHP lignin, (h) poplar 2nd-stage Cu-AHP lignin, (i) poplar CELF lignin, and (j) poplar [Ch][Lys] lignin. All NMR spectra were recorded in DMSO-*d*_6_ solvent and signals at δ = 2.5 ppm for ^1^H NMR and δ = 39.50 ppm for ^13^C NMR were considered as reference peaks to assign the 2D (HSQC) NMR signals. **Figure S5.** Comparison of S/G ratios as determined by quantitative thioacidolysis versus (semi-quantitative) 2-D HSQC NMR.

## Data Availability

The datasets used and/or analyzed during the current study are available from the corresponding authors on reasonable request.

## References

[1] Qureshi N, Hodge D, Vertes A (2014). Biorefineries: integrated biochemical processes for liquid biofuels.

[2] Farrell AE, Plevin RJ, Turner BT, Jones AD, O’Hare M, Kammen DM (2006). Ethanol can contribute to energy and environmental goals. Science.

[3] Warner E, Schwab A, Bacovsky D. 2016 survey of non-starch alcohol and renewable hydrocarbon biofuels producers. NREL Technical Report NREL/TP-6A10-67539; 2017.

[4] Lynd LR, Liang X, Biddy MJ, Allee A, Cai H, Foust T, Himmel ME, Laser MS, Wang M, Wyman CE (2017). Cellulosic ethanol: status and innovation. Curr Opin Biotechnol.

[5] Lautala PT, Hilliard MR, Webb E, Busch I, Richard Hess J, Roni MS, Hilbert J, Handler RM, Bittencourt R, Valente A, Laitinen T (2015). Opportunities and challenges in the design and analysis of biomass supply chains. Environ Manag.

[6] Langholtz M, Stokes B, Eaton L. 2016 billion-ton report: advancing domestic resources for a thriving bioeconomy. ORNL Technical Report ORNL/TM-2016/160; 2016.

[7] Taylor G, Allwright MR, Smith H, Polle A, Wildhagen H, Hertzberg M, Bhalerao R, Keurentjes JJ, Scalabrin S, Scaglione D (2016). Bioenergy trees: genetic and genomic strategies to improve yield. Perennial biomass crops for a resource-constrained world.

[8] Hansen E, Moore L, Netzer D, Ostry M, Phipps H, Zavitkovski J (1983) Establishing intensively cultured hybrid poplar plantations for fuel and fiber. USDA-FS North Central Forest Experiment Station Technical Report NC-78; 1983.

[9] Zamora DS, Apostol KG, Berguson WE, Volk TA, Wright J, Ogdahl EJ, Jose S, Bhaskar T (2015). Short rotation woody crops biomass production. Biomass and biofuels: advanced biorefineries for sustainable production and distribution.

[10] Hansen EA, Ostry ME, Johnson WD, Tolsted DN, Netzer DA, Berguson WE, Hall RB (1994) Field performance of *Populus* in short-rotation intensive culture plantations in the north-central U.S. USDA-FS North Central Forest Experiment Station Technical Report NC-320; 1994.

[11] Pan X, Gilkes N, Kadla J, Pye K, Saka S, Gregg D, Ehara K, Xie D, Lam D, Saddler J (2006). Bioconversion of hybrid poplar to ethanol and co-products using an organosolv fractionation process: optimization of process yields. Biotechnol Bioeng.

[12] Wyman CE, Dale BE, Elander RT, Holtzapple M, Ladisch MR, Lee Y, Mitchinson C, Saddler JN (2009). Comparative sugar recovery and fermentation data following pretreatment of poplar wood by leading technologies. Biotechnol Progr.

[13] Miller RO (2018). Growth variation among hybrid poplar varieties in Michigan, USA and the implications for commercial biomass production. Bioenergy Res.

[14] Ledford H (2014). Brazil considers transgenic trees. Nature.

[15] Turnbull JW, Boyle JR, Winjum JR, Kavanagh K, Jensen EC (1999). Eucalypt plantations. Planted forests: contributions to the quest for sustainable societies.

[16] Hinchee M, Rottmann W, Mullinax L, Zhang C, Chang S, Cunningham M, Pearson L, Nehra N, Tomes D, Lakshmanan P, Songstad D (2011). Short-rotation woody crops for bioenergy and biofuels applications. Biofuels: global impact on renewable energy, production agriculture, and technological advancements.

[17] Hodge DB, Karim MN, Schell DJ, McMillan JD (2008). Soluble and insoluble solids contributions to high-solids enzymatic hydrolysis of lignocellulose. Bioresour Technol.

[18] Jönsson LJ, Martin C (2016). Pretreatment of lignocellulose: formation of inhibitory by-products and strategies for minimizing their effects. Bioresour Technol.

[19] Tian S, Zhu W, Gleisner R, Pan XJ, Zhu JY (2011). Comparisons of SPORL and dilute acid pretreatments for sugar and ethanol productions from aspen. Biotechnol Progr.

[20] Farías-Sánchez JC, López-Miranda J, Castro-Montoya A, Saucedo-Luna J, Carrillo-Parra A, López-Albarrán P, Pineda-Pimentel MG, Rutiaga-Quiñones JG (2015). Comparison of five pretreatments for the production of fermentable sugars obtained from *Pinus pseudostrobus* L. wood. EXCLI J.

[21] Laser M, Schulman D, Allen SG, Lichwa J, Antal MJ, Lynd LR (2002). A comparison of liquid hot water and steam pretreatments of sugar cane bagasse for bioconversion to ethanol. Bioresour Technol.

[22] Wyman CE, Dale BE, Elander RT, Holtzapple M, Ladisch MR, Lee YY (2005). Coordinated development of leading biomass pretreatment technologies. Bioresour Technol.

[23] Elander RT, Dale BE, Holtzapple M, Ladisch MR, Lee YY, Mitchinson C, Saddler JN, Wyman CE (2009). Summary of findings from the biomass refining consortium for applied fundamentals and innovation (CAFI): corn stover pretreatment. Cellulose.

[24] Wyman CE, Balan V, Dale BE, Elander RT, Falls M, Hames B, Holtzapple MT, Ladisch MR, Lee YY, Mosier N, Pallapolu VR, Shi J, Thomas SR, Warner RE (2011). Comparative data on effects of leading pretreatments and enzyme loadings and formulations on sugar yields from different switchgrass sources. Bioresour Technol.

[25] Singh S, Cheng G, Sathitsuksanoh N, Wu D, Varanasi P, George A, Balan V, Gao X, Kumar R, Dale BE, Wyman CE, Simmons BA (2015). Comparison of different biomass pretreatment techniques and their impact on chemistry and structure. Front Energy Res.

[26] Gao X, Kumar R, Singh S, Simmons BA, Balan V, Dale BE, Wyman CE (2014). Comparison of enzymatic reactivity of corn stover solids prepared by dilute acid, AFEX, and ionic liquid pretreatments. Biotechnol Biofuels.

[27] Uppugundla N, Sousa LD, Chundawat SPS, Yu XR, Simmons B, Singh S, Gao XD, Kumar R, Wyman CE, Dale BE, Balan V (2014). A comparative study of ethanol production using dilute acid, ionic liquid and AFEX™ pretreated corn stover. Biotechnol Biofuels.

[28] Nguyen TY, Cai CM, Kumar R, Wyman CE (2015). Co-solvent pretreatment reduces costly enzyme requirements for high sugar and ethanol yields from lignocellulosic biomass. Chemsuschem.

[29] Smith MD, Mostofian B, Cheng X, Petridis L, Cai CM, Wyman CE, Smith JC (2016). Cosolvent pretreatment in cellulosic biofuel production: effect of tetrahydrofuran-water on lignin structure and dynamics. Green Chem.

[30] Nguyen TY, Cai CM, Osman O, Kumar R, Wyman CE (2016). CELF pretreatment of corn stover boosts ethanol titers and yields from high solids SSF with low enzyme loadings. Green Chem.

[31] Cai CM, Zhang T, Kumar R, Wyman CE (2013). THF co-solvent enhances hydrocarbon fuel precursor yields from lignocellulosic biomass. Green Chem.

[32] Nguyen TY, Cai CM, Kumar R, Wyman CE (2017). Overcoming factors limiting high-solids fermentation of lignocellulosic biomass to ethanol. Proc Nat Acad Sci.

[33] Hou XD, Smith TJ, Li N, Zong MH (2012). Novel renewable ionic liquids as highly effective solvents for pretreatment of rice straw biomass by selective removal of lignin. Biotechnol Bioeng.

[34] Sun N, Parthasarathi R, Socha AM, Shi J, Zhang S, Stavila V, Sale KL, Simmons BA, Singh S (2014). Understanding pretreatment efficacy of four cholinium and imidazolium ionic liquids by chemistry and computation. Green Chem.

[35] Socha AM, Parthasarathi R, Shi J, Pattathil S, Whyte D, Bergeron M, George A, Tran K, Stavila V, Venkatachalam S (2014). Efficient biomass pretreatment using ionic liquids derived from lignin and hemicellulose. Proc Nat Acad Sci..

[36] Dutta T, Shi J, Sun J, Zhang X, Cheng G, Simmons BA, Singh S, Rafal-Lukasik R (2015). Ionic liquid pretreatment of lignocellulosic biomass for biofuels and chemicals. Ionic liquids in the biorefinery concept: challenges and perspectives.

[37] Parthasarathi R, Sun J, Dutta T, Sun N, Pattathil S, Konda NM, Peralta AG, Simmons BA, Singh S (2016). Activation of lignocellulosic biomass for higher sugar yields using aqueous ionic liquid at low severity process conditions. Biotechnol Biofuels.

[38] Li Z, Chen CH, Liu T, Mathrubootham V, Hegg EL, Hodge DB (2013). Catalysis with Cu^II^(bpy) improves alkaline hydrogen peroxide pretreatment. Biotechnol Bioeng.

[39] Li Z, Bansal N, Azarpira A, Bhalla A, Chen C, Ralph J, Hegg EL, Hodge DB (2015). Chemical and structural changes associated with Cu-catalyzed alkaline oxidative pretreatment of hybrid poplar. Biotechnol Biofuels.

[40] Li Z, Chen CH, Hegg EL, Hodge DB (2013). Rapid and effective oxidative pretreatment of woody biomass at mild reaction conditions and low oxidant loadings. Biotechnol Biofuels.

[41] Bhalla A, Bansal N, Stoklosa RJ, Fountain M, Ralph J, Hodge DB, Hegg EL (2016). Effective alkaline metal-catalyzed oxidative delignification of hybrid poplar. Biotechnol Biofuels.

[42] Bansal N, Bhalla A, Pattathil S, Adelman SL, Hahn MG, Hodge DB, Hegg EL (2016). Cell wall-associated transition metals improve alkaline-oxidative pretreatment in diverse hardwoods. Green Chem.

[43] Ong RG, Chundawat SP, Hodge DB, Keskar S, Dale BE, McCann MC, Buckeridge MS, Carpita NC (2014). Linking plant biology and pretreatment: understanding the structure and organization of the plant cell wall and interactions with cellulosic biofuel production. Plants and BioEnergy.

[44] Stoklosa RJ, Hodge DB (2015). Fractionation and improved enzymatic deconstruction of hardwoods with alkaline delignification. Bioenergy Res.

[45] Stoklosa RJ, Hodge DB (2012). Extraction, recovery, and characterization of hardwood and grass hemicelluloses for integration into biorefining processes. Ind Eng Chem Res.

[46] Smith MD, Cai CM, Cheng X, Petridis L, Smith JC (2018). Temperature-dependent phase behaviour of tetrahydrofuran–water alters solubilization of xylan to improve co-production of furfurals from lignocellulosic biomass. Green Chem.

[47] Shi J, Gladden JM, Sathitsuksanoh N, Kambam P, Sandoval L, Mitra D, Zhang S, George A, Singer SW, Simmons BA, Singh S (2013). One-pot ionic liquid pretreatment and saccharification of switchgrass. Green Chem.

[48] Valette N, Perrot T, Sormani R, Gelhaye E, Morel-Rouhier M (2017). Antifungal activities of wood extractives. Fungal Biol Rev.

[49] Yeh TF, Chang MJ, Chang WJ (2014). Comparison of dilute acid and sulfite pretreatments on *Acacia confusa* for biofuel application and the influence of its extractives. J Agric Food Chem.

[50] Yang B, Wyman CE (2004). Effect of xylan and lignin removal by batch and flowthrough pretreatment on the enzymatic digestibility of corn stover cellulose. Biotechnol Bioeng.

[51] Yu Z, Jameel H, Chang H-M, Park S (2011). The effect of delignification of forest biomass on enzymatic hydrolysis. Bioresour Technol.

[52] Williams DL, Hodge DB (2014). Impacts of delignification and hot water pretreatment on the water induced cell wall swelling behavior of grasses and its relation to cellulolytic enzyme hydrolysis and binding. Cellulose.

[53] Schmidt M (2008). The Sankey diagram in energy and material flow management: part I: history. J Ind Ecol.

[54] Kalami S, Arefmanesh M, Master E, Nejad M (2017). Replacing 100% of phenol in phenolic adhesive formulations with lignin. J Appl Polym Sci.

[55] Cateto CA, Barreiro MF, Ottati C, Lopretti M, Rodrigues AE, Belgacem MN (2013). Lignin-based rigid polyurethane foams with improved biodegradation. J Cell Plast.

[56] Thielemans W, Wool RP (2005). Lignin esters for use in unsaturated thermosets: lignin modification and solubility modeling. Biomacromolecules.

[57] Sameni J, Krigstin S, Sain M (2017). Solubility of lignin and acetylated lignin in organic solvents. BioResources.

[58] Zakzeski J, Bruijnincx PC, Jongerius AL, Weckhuysen BM (2010). The catalytic valorization of lignin for the production of renewable chemicals. Chem Rev.

[59] Sun Z, Fridrich B, de Santi A, Elangovan S, Barta K (2018). Bright side of lignin depolymerization: toward new platform chemicals. Chem Rev.

[60] Deuss PJ, Scott M, Tran F, Westwood NJ, de Vries JG, Barta K (2015). Aromatic monomers by in situ conversion of reactive intermediates in the acid-catalyzed depolymerization of lignin. J Am Chem Soc.

[61] Patri AS, Mostofian B, Pu Y, Ciaffone N, Soliman M, Smith MD, Kumar R, Cheng X, Wyman CE, Tetard L, Ragauskas AJ (2019). A multifunctional co-solvent pair reveals molecular principles of biomass deconstruction. J Am Chem Soc.

[62] Meng X, Parikh A, Seemala B, Kumar R, Pu Y, Christopher P, Wyman CE, Cai CM, Ragauskas AJ (2018). Chemical transformations of poplar lignin during cosolvent enhanced lignocellulosic fractionation process. ACS Sustain Chem Eng.

[63] Ko JK, Kim Y, Ximenes E, Ladisch MR (2015). Effect of liquid hot water pretreatment severity on properties of hardwood lignin and enzymatic hydrolysis of cellulose. Biotechnol Bioeng.

[64] Gomes KR, Chimphango AF, Görgens JF (2015). Modifying solubility of polymeric xylan extracted from *Eucalyptus grandis* and sugarcane bagasse by suitable side chain removing enzymes. Carbohydr Polym.

[65] Linder Å, Bergman R, Bodin A, Gatenholm P (2003). Mechanism of assembly of xylan onto cellulose surfaces. Langmuir.

[66] Baumberger S, Abaecherli A, Fasching M, Gellerstedt G, Gosselink R, Hortling B, Li J, Saake B, de Jong E (2007). Molar mass determination of lignins by size-exclusion chromatography: towards standardisation of the method. Holzforschung.

[67] Jacobs A, Dahlman O (2001). Characterization of the molar masses of hemicelluloses from wood and pulps employing size exclusion chromatography and matrix-assisted laser desorption ionization time-of-flight mass spectrometry. Biomacromolecules.

[68] Goring D, Timell T (1960). Molecular properties of six 4-*O*-methylglucuronoxylans. J Phys Chem.

[69] Ralph J, Lundquist K, Brunow G, Lu F, Kim H, Schatz PF, Marita JM, Hatfield RD, Ralph SA, Christensen JH, Boerjan W (2004). Lignins: natural polymers from oxidative coupling of 4-hydroxyphenyl-propanoids. Phytochem Rev.

[70] Yan N, Zhao C, Dyson PJ, Wang C, Liu LT, Kou Y (2008). Selective degradation of wood lignin over noble-metal catalysts in a two-step process. Chemsuschem.

[71] Phongpreecha T, Hool NC, Stoklosa RJ, Klett AS, Foster CE, Bhalla A, Holmes D, Thies MC, Hodge DB (2017). Predicting lignin depolymerization yields from quantifiable properties using fractionated biorefinery lignins. Green Chem.

[72] Crestini C, Melone F, Sette M, Saladino R (2011). Milled wood lignin: a linear oligomer. Biomacromolecules.

[73] Ratnaweera DR, Saha D, Pingali SV, Labbé N, Naskar AK, Dadmun M (2015). The impact of lignin source on its self-assembly in solution. RSC Adv.

[74] Shi J, Thompson VS, Yancey NA, Stavila V, Simmons BA, Singh S (2013). Impact of mixed feedstocks and feedstock densification on ionic liquid pretreatment efficiency. Biofuels.

[75] Sluiter A, Hames B, Ruiz R, Scarlata C, Sluiter J, Templeton D, Crocker D. Determination of structural carbohydrates and lignin in biomass. NREL Technical Report NREL/TP-510-42618; 2008.

[76] Xu F, Sun J, Konda NM, Shi J, Dutta T, Scown CD, Simmons BA, Singh S (2016). Transforming biomass conversion with ionic liquids: process intensification and the development of a high-gravity, one-pot process for the production of cellulosic ethanol. Energy Environ Sci.

[77] Dutta T, Papa G, Wang E, Sun J, Isern NG, Cort JR, Simmons BA, Singh S (2018). Characterization of lignin streams during bionic liquid-based pretreatment from grass, hardwood, and softwood. ACS Sustain Chem Eng.

[78] Gu F, Wu W, Wang Z, Yokoyama T, Jin Y, Matsumoto Y (2015). Effect of complete dissolution in LiCl/DMSO on the isolation and characteristics of lignin from wheat straw internode. Ind Crop Prod.

[79] Bär J, Phongpreecha T, Singh SK, Yilmaz MK, Foster CE, Crowe JD, Hodge DB (2018). Deconstruction of hybrid poplar to monomeric sugars and aromatics using ethanol organosolv fractionation. Biomass Convers Bioref.

[80] Foster CE, Martin TM, Pauly M (2010). Comprehensive compositional analysis of plant cell walls (lignocellulosic biomass). Part II: carbohydrates. J Vis Exp.

[81] Malawar EG, Senak L, Wu C (2004). Introduction to size exclusion chromatography. Handbook of size exclusion chromatography and related techniques.

[82] El Hage RBN, Chrusciel L, Sanchez C, Sannigrahi P, Ragauskas A (2009). Characterization of milled wood lignin and ethanol organosolv lignin from *miscanthus*. Polym Degrad Stab.

[83] Balakshin MYCE, Santos RB, Chang H, Jameel H (2016). Structural analysis of hardwood native lignins by quantitative ^13^C NMR spectroscopy. Holzforschung.

[84] Ralph SA, Ralph J, Landucci L, Landucci L. NMR database of lignin and cell wall model compounds. U.S. Forest Products Laboratory, Madison, WI. 2009. https://www.glbrc.org/databases_and_software/nmrdatabase/NMR_DataBase_2009_Complete.pdf.

