# Supplementary material for: Performance of three delignifying pretreatments on hardwoods: hydrolysis yields, comprehensive mass balances, and lignin properties
Source: Biotechnol Biofuels. 2019 Sep 9;12:213. doi: 10.1186/s13068-019-1546-0 (PMC6732840; doi:10.1186/s13068-019-1546-0)
Supplement: Supplementary file 1 — Additional file 1: Table S1. Composition and mass yields of hybrid poplar and eucalyptus prior to and following pretreatment. Cu-AHP: Cu-catalyzed alkaline hydrogen peroxide pretreatment; [Ch][Lys]: cholinium lysinate pretreatment; CELF: co-solvent enhanced lignocellulosic fractionation. Table S2. Assignments of signals observed in 13C/1H 2D (HSQC) NMR spectra of lignins. Figure S1. Enzymatic hydrolysis xylose yields for pretreated solids of hybrid poplar (A and B) and eucalyptus (C and D) prepared by Cu-AHP, CELF, and [Ch][Lys] pretreatments as a function of enzyme loading (mg protein/g glucan in pretreated solids) and hydrolysis time. Hydrolysis was performed at a 10% (wt/vol) solids loading with the pH buffered at 5.0 for 24 or 72 h. Figure S2. Size-exclusion chromatography (SEC) elution profiles and estimated values for number average molar mass ($\bar{M}_{\text{N}}$M¯N), weight average molar mass ($\bar{M}_{\text{W}}$M¯W), and polydispersity index (PDI) for (A) eucalyptus and (B) hybrid poplar. SEC was performed using a Waters UltrahydrogelTM 250 column with a mobile phase comprising a 80:20 (v/v) solution of 0.1 M NaNO3:0.005 M NaOH/CH3CN. Figure S3. Summary of the fate of the (A, D) glucan, (B, E) xylan, and (C, F) lignin for the three pretreatments for (A, B, C) hybrid poplar and (D, E, F) eucalyptus. Hydrolysis yields are for 30 mg/g glucan enzyme loading for 72 h. Figure S4. 13C/1H 2D (HSQC) NMR spectra of (a) native cellulolytic eucalyptus lignin, (b) eucalyptus 1st-stage [file 13068_2019_1546_MOESM1_ESM.pdf]

SUPPORTING INFORMATION

Performance of Three Delignifying Pretreatments on Hardwoods: Hydrolysis Yields, Comprehensive Mass Balances, and Lignin Properties

Aditya Bhalla,[1,2] Charles M. Cai,[3,4] Feng Xu,[5] Sandip K. Singh,[6] Namita Bansal,[1,2] Thanaphong Phongpreecha,[7] Tanmoy Dutta,[5] Cliff E. Foster,[2] Rajeev Kumar,[3,4] Blake A. Simmons,[5] Seema Singh,[5] Charles E. Wyman,[3,4] Eric L. Hegg,[1,2,*] David B. Hodge[2,6,7,8,*]

1 Department of Biochemistry & Molecular Biology, Michigan State University, East Lansing, MI 48824
2 DOE Great Lakes Bioenergy Research Center (GLBRC), Michigan State University, East Lansing, MI 48824
3 Department of Chemical and Environmental Engineering, University of California, Riverside, CA
4 BioEnergy Science Center (BESC) and Center for Bioenergy Innovation (CBI), Oak Ridge National Laboratory, Oak Ridge, TN 37831
5 Joint BioEnergy Institute (JBEI), Lawrence Berkeley National Laboratory, Berkeley, CA, 94720
6 Chemical & Biological Engineering Department, Montana State University, Bozeman, MT, 50715
7 Department of Chemical Engineering and Materials Science, Michigan State University, East Lansing, MI 48824
8 Division of Sustainable Process Engineering, Luleå University of Technology, Luleå, Sweden

* Corresponding author. Email: david.hodge3@montana.edu; erichegg@msu.edu

CONTENTS **page**

Table S1 . . . . S2

Table S2 . . . . S3

Figure S1 . . . . S5

Figure S2 . . . . S6

Figure S3 . . . . S7

Figure S4 . . . . S8

Figure S5 . . . . S10

	Pretreatment	**Glucan** (% by mass)	**Xylan** (% by mass)	**Klason Lignin** (% by mass)	**Other or Unquantified** (% by mass)	**Mass Yield** (% by mass)
**Hybrid Poplar**	Untreated	44.0	15.6	24.0	16.4	100.0
	Cu-AHP 1st Stage	61.5	13.5	19.5	5.5	70.2
	Cu-AHP 2nd Stage	67.5	14.2	10.8	7.5	59.4
	[Ch][Lys]	61.1	8.8	17.8	12.2	67.8
	CELF	90.5	2.5	7.1	0.0	46.8
**Eucalyptus**	Untreated	32.8	14.4	30.0	22.8	100.0
	Cu-AHP 1st Stage	54.0	16.3	20.4	8.4	63.0
	Cu-AHP 2nd Stage	59.9	11.7	19.8	8.7	54.0
	[Ch][Lys]	55.6	8.6	22.5	13.3	53.4
	CELF	79.2	3.1	11.4	6.3	37.1

Table S1. Composition and mass yields of hybrid poplar and eucalyptus prior to and following pretreatment. Cu-AHP: Cu-catalyzed alkaline hydrogen peroxide pretreatment; [Ch][Lys]: cholinium lysinate pretreatment; CELF: co-solvent enhanced lignocellulosic fractionation.

δ_C/δ_H (ppm) lignin samples										Assignments
**Eucalyptus**					**Hybrid Poplar**					
a	b	c	d	e	f	g	h	i	j	
53.50 /3.03	53.50 /3.02	53.50 /3.02	53.53 /3.04	52.99 /2.99	53.61 /3.03	53.20 /3.03	53.20 /3.03	53.26 /3.04	52.84 /2.94	C_β–H_β in phenylcoumaran (β-5')
55.62 /3.71	55.70 /3.73	55.48 /3.70	55.65 /3.63	55.63 /3.69	55.58 /3.71	55.35 /3.72	55.57 /3.74	55.28 /3.72	55.41 /3.71	C–H in methoxy
59.87 /3.18	59.90 /3.18	59.44 /3.35	59.27 /3.24	59.16 /3.14	59.49 /3.35	59.06 /3.35	59.28 /3.37	59.42 /3.62	59.08 /3.35	Cγ–Hγ in β-O-4'
59.78 /3.62	59.75 /3.61	59.44 /3.68	59.71 /3.63	-	59.72 /3.62	59.51 /3.59	59.35 /3.71	-	59.30 /3.69	
-	62.94 /3.84	62.49 /3.38	-	63.06 /3.85	-	62.82 /3.85	-	-	62.88 /3.86	Cγ–Hγ in resinol (β-β')
71.03 /3.78	70.71 /3.74	71.17 /3.79	71.07 /3.77	-	71.01 /3.78	70.98 /3.73	71.18 /3.78	70.62 /3.77	-	Cγ–Hγ in phenylcoumaran (β-5')
71.08 /4.15	70.71 /4.13	71.02 /4.15	70.97 /4.16	-	-	70.70 /4.14	-	-	-	
71.75 /4.82	71.62 /4.82	72.08 /4.83	71.65 /4.79	-	71.75 /4.82	71.48 /4.83	71.62 /4.84	70.79 /4.72	71.63 /4.84	Cα–Hα in β-O-4'
83.27 /4.28	-	83.66 /4.25	83.35 /4.27	-	83.81 /4.26	83.30 /4.26	83.36 /4.30	83.29 /4.27	-	C_β–H_β in β-O-4' linked to a guaiacyl unit
-	85.03 /4.58	84.87 /4.62	85.0 /4.61	-	-	84.70 /4.62	84.79 /4.65	84.61 /4.60	-	Cα–Hα in β-5'
85.94 /4.07	85.94 /4.07	85.94 /4.07	85.90 /4.09	-	85.96 /4.08	85.46 /4.10	85.72 /4.10	85.49 /4.10	-	Cβ–Hβ in β-O-4' linked to a syringyl unit
-	-	-	-	-	-	86.53 /5.45	86.71 /5.45	86.63 /5.42	-	Cα–Hα in resinol (β-β')
103.89 /6.67	103.30 /6.58	103.96 /6.68	103.23 /6.59	103.96 /6.67	103.89 /6.67	103.70 /6.67	103.86 /6.69	103.09 /6.60	103.68 /6.70	$C_{2,6}$–$H_{2,6}$ in syringyl units (*S*)
106.14 /7.04	106.60 /7.19	106.50 /7.19	106.30 /7.30	106.17 /7.29	106.09 /7.03	105.91 /7.29	106.36 /7.20	105.98 /7.30	-	$C_{2,6}$–$H_{2,6}$ in oxidized syringyl units (*S*')
106.2 /7.22	106.09 /7.29	106.19 /7.29	106.81 /7.20	106.85 /6.48	106.02 /7.22	106.50 /7.20	-	106.46 /7.13	-	

110.81 /6.94	109.24 /7.10	110.86 /6.95	111.01 /6.98	110.99 /6.95	110.97 /6.98	110.67 /6.96	110.92 /6.97	110.76 /6.91	110.73 /6.98	C_2–H_2 in guaiacyl units (*G*)
-	-	-	110.23 /7.36	112.96 /7.19	-	-	111.85 /7.37	-	-	C_2–H_2 in oxidized guaiacyl units (*G'*)
114.55 /6.67	115.02 /6.67	114.92 /6.93	115.09 /6.73	114.61 /6.69	114.96 /6.76	114.29 /6.67	114.40 /6.69	114.73 /6.77	114.35 /6.73	$C_{3,5}$–$H_{3,5}$ in p-benzoate units (PB)
114.86 /6.92	114.95 /6.82	114.75 /6.92	115.15 /6.94	114.81 /6.92	114.69 /6.90	114.82 /6.95	114.96 /6.96	114.69 /6.87	114.78 /6.94	C_5–H_5 in guaiacyl units (*G*)
118.86 /6.75	117.97 /6.59	118.99 /6.80	118.74 /6.82	119.02 /6.77	118.90 /6.74	118.65 /6.77	118.86 /6.82	118.36 /6.75	118.74 /6.8	C_6–H_6 in guaiacyl units (*G*)
-	128.63 /7.20	-	-	128.43 /7.20	-	-	-	-	-	$C_{2,6}$–$H_{2,6}$ in *p*-hydroxylphenyl units (*H*)
131.47 /7.67	-	-	-	-	131.26 /7.64	131.02 /7.77	-	130.91 /7.64	130.92 /7.66	$C_{2,6}$–$H_{2,6}$ in *p*-benzoate units (PB)

Note: (-) not detected. (a) native lignin eucalyptus, (b) Pre-extraction eucalyptus, (c) Cu-AHP eucalyptus, (d) CELF eucalyptus, (e) [Ch][Lys] eucalyptus, (f) native lignin poplar, (g) Pre-extraction poplar, (h) Cu-AHP poplar, (i) CELF poplar, (j) [Ch][Lys] poplar

Table S2. Assignments of signals observed in $^{13}C/^{1}H$ 2D (HSQC) NMR spectra of lignins.

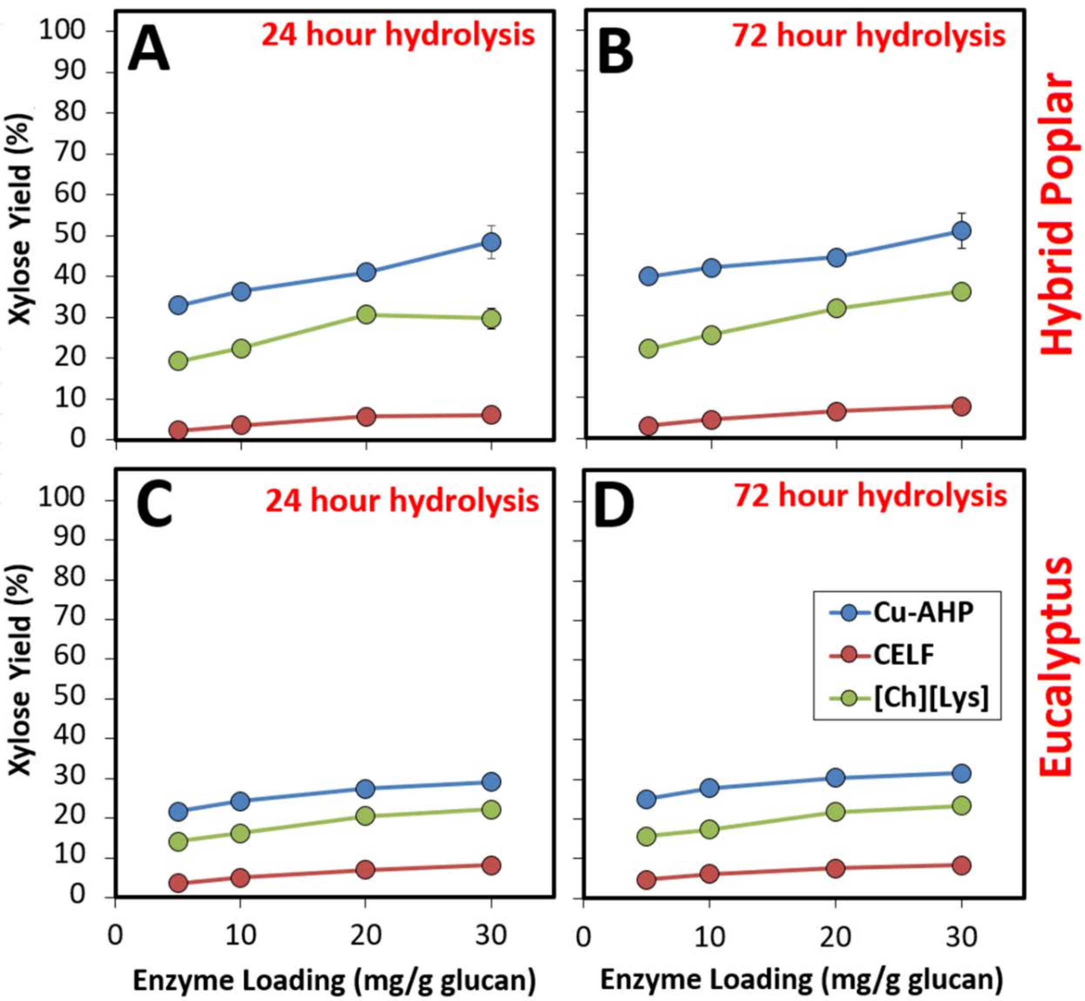

Figure S1: Enzymatic hydrolysis xylose yields for pretreated solids of hybrid poplar (**A** and **B**) and eucalyptus (**C** and **D**) prepared by Cu-AHP, CELF, and [Ch][Lys] pretreatments as a function of enzyme loading (mg protein/g glucan in pretreated solids) and hydrolysis time. Hydrolysis was performed at a 10% (wt/vol) solids loading with the pH buffered at 5.0 for 24 or 72 h.

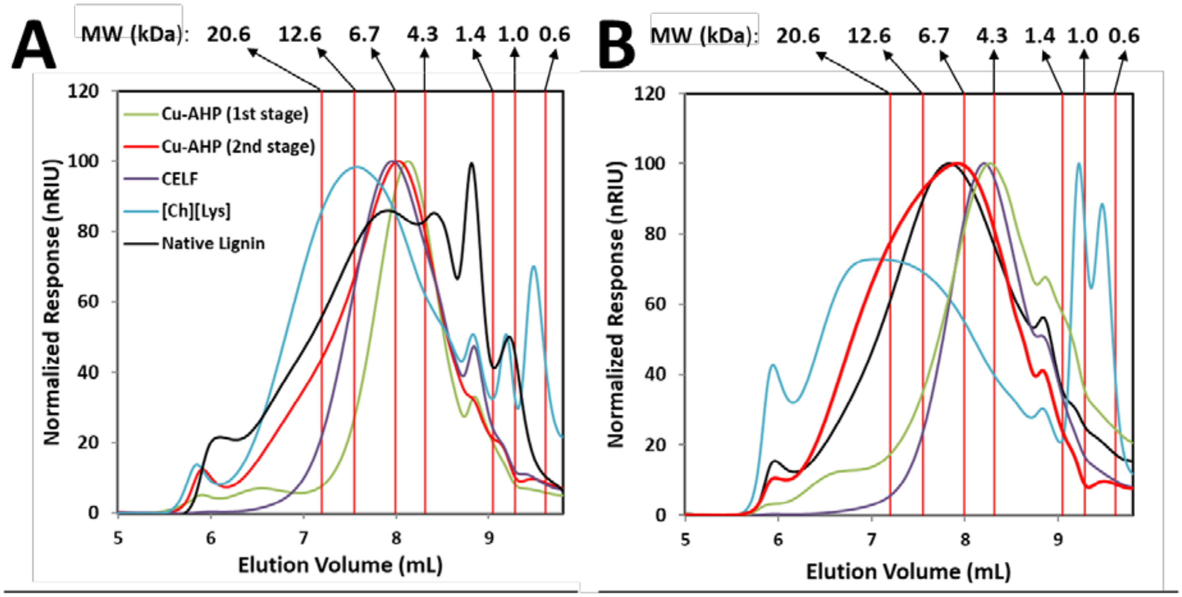

Lignin	M_N	M_W	PDI
Cu-AHP (1st stage)	6,000	9,100	1.5
Cu-AHP (2nd stage)	12,400	27,300	2.2
CELF	7,500	12,600	1.7
[Ch][Lys]	13,158	29,390	2.2
Native lignin	12,470	43,400	3.5

Eucalyptus

Lignin	M_N	M_W	PDI
Cu-AHP (1st stage)	5,800	13,000	2.2
Cu-AHP (2nd stage)	16,800	43,200	2.6
CELF	5,200	8,500	1.6
[Ch][Lys]	18,100	41,400	2.3
Native lignin	15,100	48,400	3.2

Hybrid Poplar

Figure S2: Size exclusion chromatography (SEC) elution profiles and estimated values for number average molar mass ($\overline{M}_N$), weight average molar mass ($\overline{M}_W$), and polydispersity index (PDI) for **(A)** eucalyptus and **(B)** hybrid poplar. SEC was performed using a Waters Ultrahydrogel™ 250 column with a mobile phase comprising a 80:20 (v/v) solution of 0.1 M $NaNO_3$:0.005 M $NaOH/CH_3CN$.

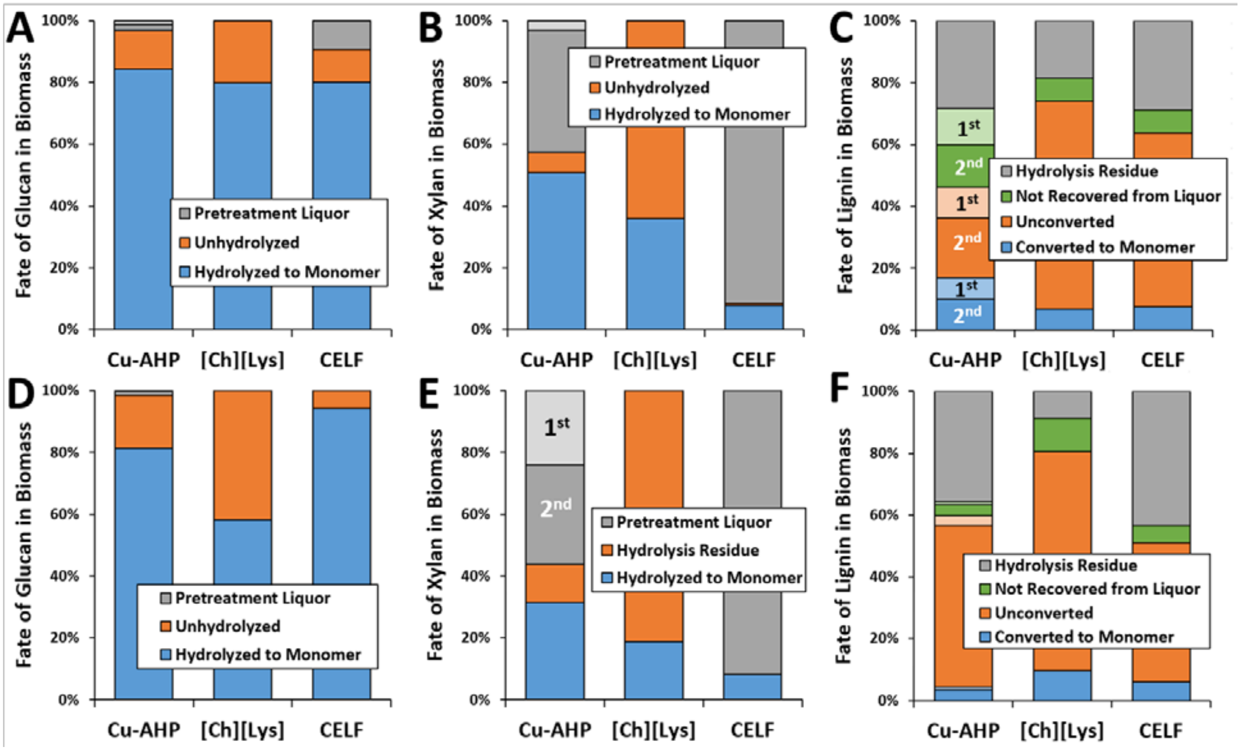

Figure S3: Summary of the fate of the (A, D) glucan, (B, E) xylan, and (C, F) lignin for the three pretreatments for (A, B, C) hybrid poplar and (D, E, F) eucalyptus. Hydrolysis yields are for 30 mg/g glucan enzyme loading for 72 h.

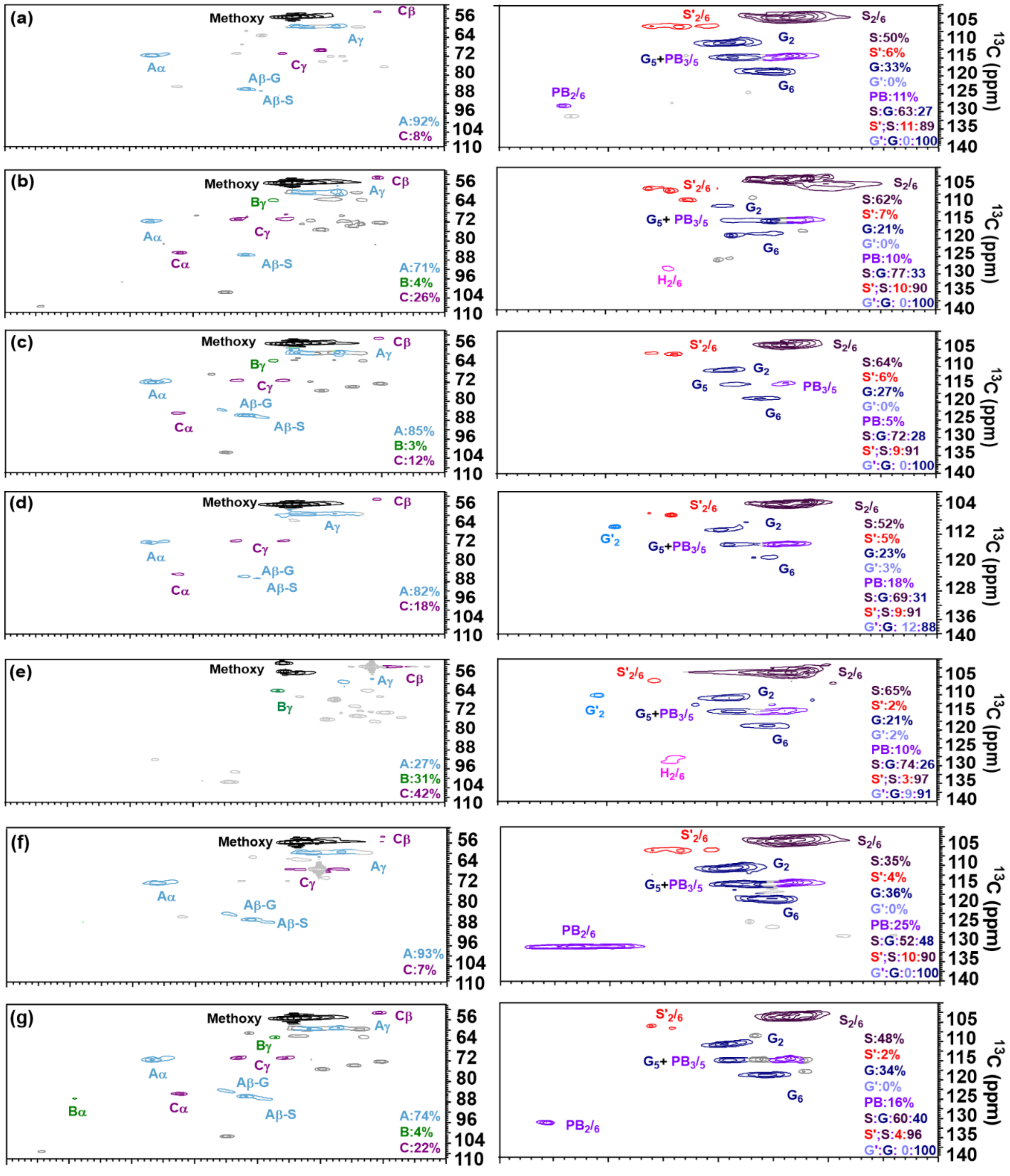

(a)
Methoxy
Cβ
Aγ
Aα
Cγ
Aβ-G
Aβ-S
A:92%
C:8%
56 64 72 80 88 96 104 110
$S'_{2/6}$
$S_{2/6}$
G_2
G_5+$PB_{3/5}$
G_6
$PB_{2/6}$
S:50%
S':6%
G:33%
G':0%
PB:11%
S:G:63:27
S';S:11:89
G':G:0:100
105 110 115 120 125 130 135 140
^{13}C (ppm)
(b)
Methoxy
Cβ
Aγ
Bγ
Aα
Cγ
Cα
Aβ-S
A:71%
B:4%
C:26%
56 64 72 80 88 96 104 110
$S'_{2/6}$
$S_{2/6}$
G_2
G_5+ $PB_{3/5}$
G_6
$H_{2/6}$
S:62%
S':7%
G:21%
G':0%
PB:10%
S:G:77:33
S';S:10:90
G':G: 0:100
105 110 115 120 125 130 135 140
^{13}C (ppm)
(c)
Methoxy
Cβ
Aγ
Bγ
Aα
Cγ
Aβ-G
Cα
Aβ-S
A:85%
B:3%
C:12%
56 64 72 80 88 96 104 110
$S'_{2/6}$
$S_{2/6}$
G_2
G_5
$PB_{3/5}$
G_6
S:64%
S':6%
G:27%
G':0%
PB:5%
S:G:72:28
S';S:9:91
G':G: 0:100
105 110 115 120 125 130 135 140
^{13}C (ppm)
(d)
Methoxy
Cβ
Aγ
Aα
Cγ
Aβ-G
Cα
Aβ-S
A:82%
C:18%
56 64 72 80 88 96 104 110
$S'_{2/6}$
$S_{2/6}$
G'_2
G_2
G_5+$PB_{3/5}$
G_6
S:52%
S':5%
G:23%
G':3%
PB:18%
S:G:69:31
S';S:9:91
G':G: 12:88
104 112 120 128 136 140
^{13}C (ppm)
(e)
Methoxy
Aγ
Cβ
Bγ
A:27%
B:31%
C:42%
56 64 72 80 88 96 104 110
$S'_{2/6}$
$S_{2/6}$
G_2
G'_2
G_5+$PB_{3/5}$
G_6
$H_{2/6}$
S:65%
S':2%
G:21%
G':2%
PB:10%
S:G:74:26
S';S:3:97
G':G:9:91
105 110 115 120 125 130 135 140
^{13}C (ppm)
(f)
Methoxy
Cβ
Aγ
Cγ
Aα
Aβ-G
Aβ-S
A:93%
C:7%
56 64 72 80 88 96 104 110
$S'_{2/6}$
$S_{2/6}$
G_2
G_5+$PB_{3/5}$
G_6
$PB_{2/6}$
S:35%
S':4%
G:36%
G':0%
PB:25%
S:G:52:48
S';S:10:90
G':G:0:100
105 110 115 120 125 130 135 140
^{13}C (ppm)
(g)
Methoxy
Cβ
Aγ
Bγ
Cγ
Aα
Aβ-G
Bα
Cα
Aβ-S
A:74%
B:4%
C:22%
56 64 72 80 88 96 104 110
$S'_{2/6}$
$S_{2/6}$
G_2
G_5+ $PB_{3/5}$
G_6
$PB_{2/6}$
S:48%
S':2%
G:34%
G':0%
PB:16%
S:G:60:40
S';S:4:96
G':G: 0:100
105 110 115 120 125 130 135 140
^{13}C (ppm)

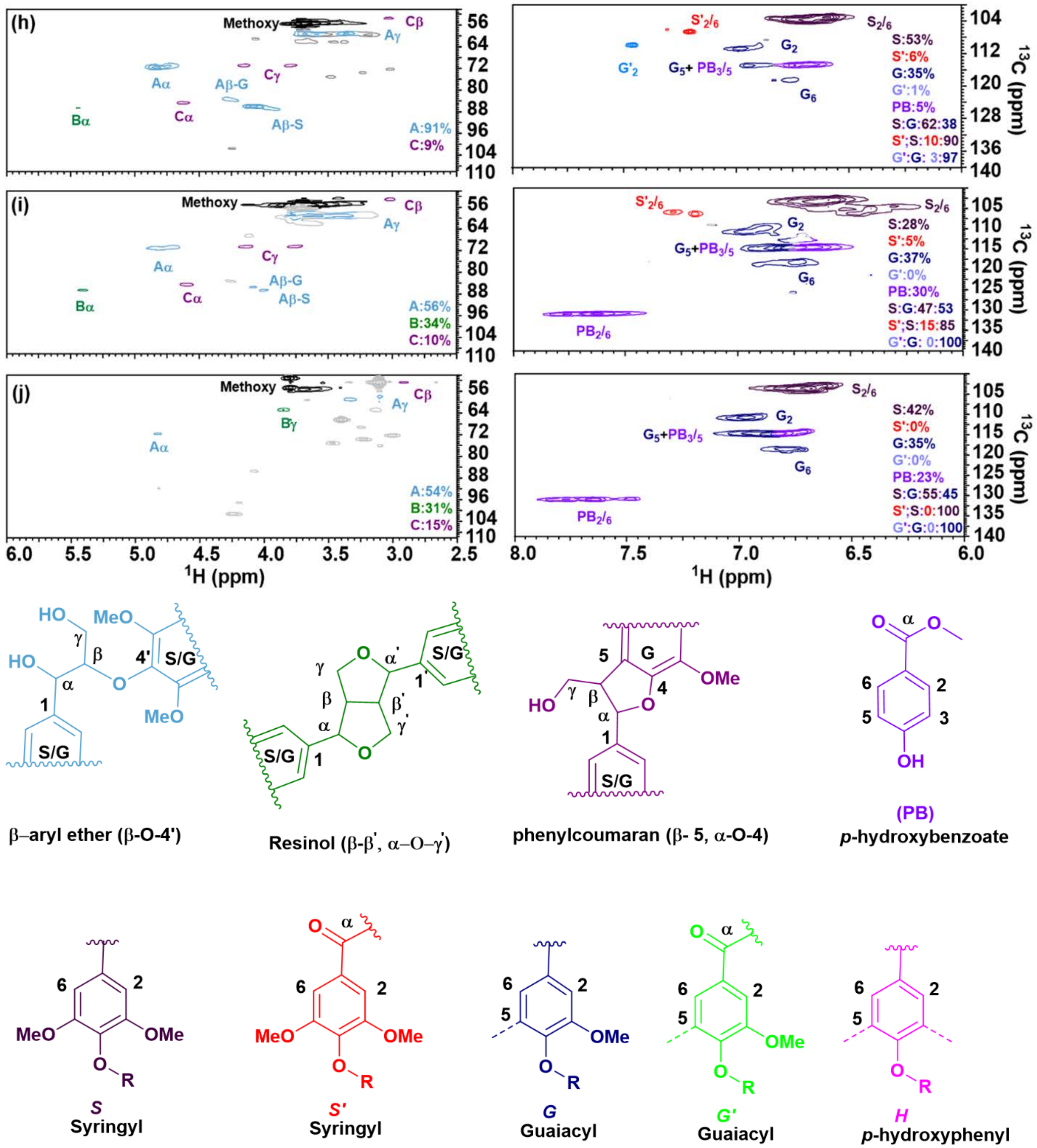

Figure S4: $^{13}C/^{1}H$ 2D (HSQC) NMR spectra of (a) native cellulolytic eucalyptus lignin, (b) eucalyptus 1st-stage Cu-AHP lignin, (c) eucalyptus 2nd-stage Cu-AHP lignin, (d) eucalyptus CELF lignin, (e) eucalyptus [Ch][Lys] lignin, (f) native cellulolytic poplar lignin, (g) poplar 1st-stage Cu-AHP lignin, (h) poplar 2nd-stage Cu-AHP lignin, (i) poplar CELF lignin, and (j) poplar [Ch][Lys] lignin. All NMR spectra were recorded in DMSO-d_6 solvent and signals at δ= 2.5 ppm for ^{1}H NMR and δ= 39.50 ppm for ^{13}C NMR were considered as reference peaks to assign the 2D (HSQC) NMR signals.

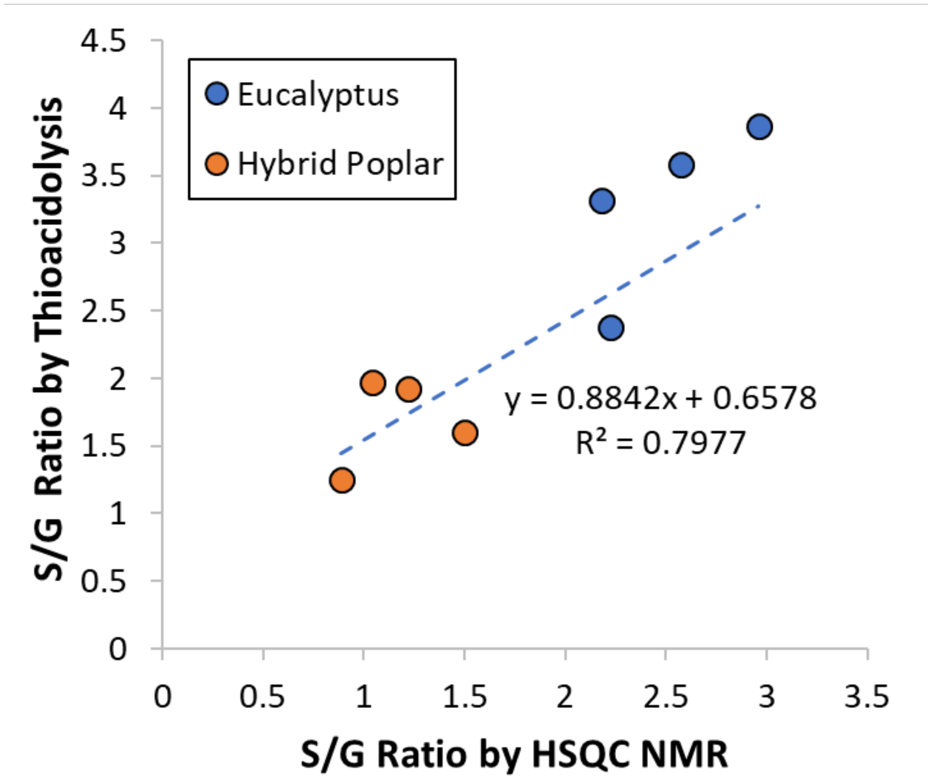

Figure S5: Comparison of S/G ratios as determined by quantitative thioacidolysis versus (semi-quantitative) 2-D HSQC NMR.
